# Supplementary material for: Using nutritional survey data to inform the design of sugar-sweetened beverage taxes in low-resource contexts: a cross-sectional analysis based on data from an adult Caribbean population
Source: BMJ Open. 2020 Sep 10;10(9):e035981. doi: 10.1136/bmjopen-2019-035981 (PMC7485232; doi:10.1136/bmjopen-2019-035981)
Supplement: Supplementary data [file bmjopen-2019-035981supp001.pdf]

Appendix Files:

1. **Appendix Table 1:** Beverage category definitions
2. **Appendix Table 2:** STROBE-nut: An extension of the STROBE statement for nutritional epidemiology
3. **Appendix Table 3:** Products by taxable status and free sugar concentration levels (with specific brands as exemplars)
4. **Appendix Text 1:** Definition of beverage categories and nutrient composition
5. **Appendix Text 2:** Sugar concentration by product types
6. **Appendix Figure 1:** Distribution of SSB consumption in mean daily servings/capita, given any SSB consumption, Barbados 2012-2013: the Barbados Salt Intake Study
7. **Appendix Figure 2**: Per-person mean Total Energy Intake (TEI) attributable to SSB-related free sugars consumption (%), given any consumption, Barbados 2012-2013: the Barbados Salt Intake Study
8. **Appendix Figure 3:** Mean per-person free sugar consumed from soft drinks amongst adults aged 25-64, by product type and taxable status, Barbados 2012-2013: the Barbados Salt Intake Study

Appendix Table 1: Beverage category definitions

Main Categories	Taxable status	Level I Category	Level II Categories	Examples of frequently consumed drinks
SSBs	Taxed SSBs	Soda	Soda	Coca Cola, Frutee, Fanta, Sprite, Pepsi
		Juice Drinks	Juice Drinks	Pinehill Dairy Juice drinks, Fruta
		Energy/Sports/Malt Drinks	Malt Drinks	VitaMalt
			Energy Drinks	Redbull, Monster
			Sports Drinks	Gatorade, Lucozade
		Other taxed SSBs	Flavored SSB water	Cranwater
			Flavoured Dairy	Indulgence Milk
			Other taxed SSBs	Store-bought iced tea, Ensure, Supligen, seamoss
	Untaxed SSBs	Mauby*	Mauby*	Mauby*
		Powdered Juice Drinks	Powdered Juice Drinks	Tang, turbo, koolaid
		Hot Chocolate	Powdered hot chocolate	Milo, Nestle
		Sweetened tea/coffee	Homemade sweetened tea/coffee	coffee, tea or iced tea w/ added sugar or sweetened condensed milk
		Sweetened condensed milk	Sweetened condensed milk	Sweetened condensed milk consumed with cereal or cream of wheat as a milk substitute
		Other untaxed SSBs	Homemade SSBs juice drinks	sugar-sweetened homemade smoothies
				sugar-sweetened homemade juices
			Other untaxed SSBs	snowcone, milkshake, purchased sweetened coffee drinks (lattes, mochas etc)
Non-SSBs	Untaxed non-SSBs	Water	Water	Tap water, bottled water, soda water
		NAS juice (no added sugar)	NAS Juice	NAS Pinehill Dairy, Dewlands, Ceres
			Homemade non-SSBs	no-sugar added homemade juices
				no-sugar added homemade smoothies
		Other non-SSB	no added sugar coffee/tea	Coffee/tea with no added sugar
		Milk	Milk	Milk
		ASB	Diet Soda	Diet Coke

9. *Although mauby could be considered a homemade drink it can also be purchased as a syrup or a ready-made drink, and as such we report it as a separate category under SSBs.

Appendix Table 2: STROBE-nut: An extension of the STROBE statement for nutritional epidemiology (Lachat C et al., 2016)

Item	Item nr	STROBE recommendations	Extension for Nutritional Epidemiology studies (STROBE-nut)	Reported on page #
**Title and abstract**	1	(a) Indicate the study's design with a commonly used term in the title or the abstract.<br><br>(b) Provide in the abstract an informative and balanced summary of what was done and what was found.	**nut-1**State the dietary/nutritional assessment method(s) used in the title, abstract, or keywords.	**Abstract**
**Introduction**				
Background rationale	2	Explain the scientific background and rationale for the investigation being reported.		1-2
Objectives	3	State specific objectives, including any pre-specified hypotheses.		3
**Methods**				
Study design	4	Present key elements of study design early in the paper.		3
Settings	5	Describe the setting, locations, and relevant dates, including periods of recruitment, exposure, follow-up, and data collection.	**nut-5**Describe any characteristics of the study settings that might affect the dietary intake or nutritional status of the participants, if applicable.	3
Participants	6	a) Cohort study—Give the eligibility criteria, and the sources and methods of selection of participants. Describe methods of follow-up.<br><br>Case-control study—Give the eligibility criteria, and the sources and methods of case ascertainment and control selection. Give the rationale for the choice of cases and controls.<br><br>Cross-sectional study—Give the eligibility criteria, and the sources and methods of selection of participants.<br><br>(b) Cohort study—For matched studies, give matching criteria and number of exposed and unexposed.	**nut-6** Report particular dietary, physiological or nutritional characteristics that were considered when selecting the target population.	3-4

Item	Item nr	STROBE recommendations	Extension for Nutritional Epidemiology studies (STROBE-nut)	Reported on page #
		Case-control study—For matched studies, give matching criteria and the number of controls per case.		
Variables	7	Clearly define all outcomes, exposures, predictors, potential confounders, and effect modifiers. Give diagnostic criteria, if applicable.	**nut-7.1**Clearly define foods, food groups, nutrients, or other food components.	**4-5**
			**nut-7.2** When using dietary patterns or indices, describe the methods to obtain them and their nutritional properties.	**3**
Data sources - measurements	8	For each variable of interest, give sources of data and details of methods of assessment (measurement).Describe comparability of assessment methods if there is more than one group.	**nut-8.1** Describe the dietary assessment method(s), e.g., portion size estimation, number of days and items recorded, how it was developed and administered, and how quality was assured. Report if and how supplement intake was assessed.	**3-4**
			**nut-8.2** Describe and justify food composition data used. Explain the procedure to match food composition with consumption data. Describe the use of conversion factors, if applicable.	**3-4**
			**nut-8.3** Describe the nutrient requirements, recommendations, or dietary guidelines and the evaluation approach usedto compare intake with the dietary reference values, if applicable.	**5**
			**nut-8.4** When using nutritional biomarkers, additionally use the STROBE Extension for Molecular Epidemiology (STROBE-ME). Report the type of biomarkers used and their usefulness as dietary exposure markers.	**NA**
			**nut-8.5** Describe the assessment of nondietary data (e.g., nutritional status and influencing factors) and timing of the assessment of these variables in relation to dietary assessment.	**4-5**

Item	Item nr	STROBE recommendations	Extension for Nutritional Epidemiology studies (STROBE-nut)	Reported on page #
			**nut-8.6** Report on the validity of the dietary or nutritional assessment methods and any internal or external validation used in the study, if applicable.	**3-4**
Bias	9	Describe any efforts to address potential sources of bias.	**nut-9** Report how bias in dietary or nutritional assessment was addressed, e.g., misreporting, changes in habits as a result of being measured, or data imputation from other sources	**3-4 and Appendix Text 1-2**
Study Size	10	Explain how the study size was arrived at.		**3-4**
Quantitative variables	11	Explain how quantitative variables were handled in the analyses. If applicable, describe which groupings were chosen and why.	**nut-11** Explain categorization of dietary/nutritional data (e.g., use of N-tiles and handling of nonconsumers) and the choice of reference category, if applicable.	**4**
Statistical Methods	12	(a) Describe all statistical methods, including those used to control for confounding<br><br>(b) Describe any methods used to examine subgroups and interactions.<br><br>(c) Explain how missing data were addressed.<br><br>(d) Cohort study—If applicable, explain how loss to follow-up was addressed.<br><br>Case-control study—If applicable, explain how matching of cases and controls was addressed.<br><br>Cross-sectional study—If applicable, describe analytical methods taking account of sampling strategy.<br><br>(e) Describe any sensitivity analyses.	**nut-12.1** Describe any statistical method used to combine dietary or nutritional data, if applicable.<br><br>**nut-12.2** Describe and justify the method for energy adjustments, intake modeling, and use of weighting factors, if applicable.<br><br>**nut-12.3** Report any adjustments for measurement error, i.e,. from a validity or calibration study.	**3, 4**<br><br>**3,4, 6**
**Results**				
Participants	13	(a) Report the numbers of individuals at each stage of the study—e.g., numbers potentially eligible, examined for eligibility, confirmed eligible, included	**nut-13** Report the number of individuals excluded based on missing, incomplete or implausible dietary/nutritional data.	**4**

Item	Item nr	STROBE recommendations	Extension for Nutritional Epidemiology studies (STROBE-nut)	Reported on page #
		in the study, completing follow-up, and analyzed.<br><br>(b) Give reasons for non-participation at each stage.<br><br>(c) Consider use of a flow diagram.		
Descriptive data	14	(a) Give characteristics of study participants (e.g., demographic, clinical, social) and information on exposures and potential confounders<br><br>(b) Indicate the number of participants with missing data for each variable of interest<br><br>(c) Cohort study—Summarize follow-up time (e.g., average and total amount)	**nut-14** Give the distribution of participant characteristics across the exposure variables if applicable. Specify if food consumption of total population or consumers only were used to obtain results.	**Table 1, p5**
Outcome data	15	Cohort study—Report numbers of outcome events or summary measures over time.<br><br>Case-control study—Report numbers in each exposure category, or summary measures of exposure.<br><br>Cross-sectional study—Report numbers of outcome events or summary measures.		
Main results	16	(a) Give unadjusted estimates and, if applicable, confounder-adjusted estimates and their precision (e.g., 95% confidence interval).<br><br>Make clear which confounders were adjusted for and why they were included.<br><br>(b) Report category boundaries when continuous variables were categorized.<br><br>(c) If relevant, consider translating estimates of relative risk into absolute risk for a meaningful time period.	**nut-16** Specify if nutrient intakes are reported with or without inclusion of dietary supplement intake, if applicable.	**NA**
Other analyses	17	Report other analyses done—e.g., analyses of subgroups and interactions and sensitivity analyses.	**nut-17**Report any sensitivity analysis (e.g., exclusion of misreporters or	**NA**

Item	Item nr	STROBE recommendations	Extension for Nutritional Epidemiology studies (STROBE-nut)	Reported on page #
			outliers) and data imputation, if applicable.	
**Discussion**				
Key results	18	Summarize key results with reference to study objectives.		7
Limitation	19	Discuss limitations of the study, taking into account sources of potential bias or imprecision. Discuss both direction and magnitude of any potential bias.	**nut-19** Describe the main limitations of the data sources and assessment methods used and implications for the interpretation of the findings.	**8**
Interpretation	20	Give a cautious overall interpretation of results considering objectives, limitations, multiplicity of analyses, results from similar studies, and other relevant evidence.	**nut-20** Report the nutritional relevance of the findings, given the complexity of diet or nutrition as an exposure.	**8-10**
Generalizability	21	Discuss the generalizability (external validity) of the study results.		10
**Other information**				
Funding	22	Give the source of funding and the role of the funders for the present study and, if applicable, for the original study on which the present article is based.		**16**
Ethics			**nut-22.1**Describe the procedure for consent and study approval from ethics committee(s).	**16**
Supplementary material			**nut-22.2** Provide data collection tools and data as online material or explain how they can be accessed.	**Appendix File**

Alvarado M, *et al. BMJ Open* 2020; 10:e035981. doi: 10.1136/bmjopen-2019-035981

Appendix Table 3: Products by taxable status and free sugar concentration levels (with specific brands as exemplars)

Free sugar concentration	Taxable status	Products
**1-2.9**	Taxed	NA
	Untaxed	SSB coffee & tea, soymilk
**3-4.9**	Taxed	Flavoured water (Cranwater)
	Untaxed	Hot chocolate, soy milk, SSB coffee & tea, powdered juice (Mak-C)
**5-6.9**	Taxed	Sports drinks (Powerade), soda (Frutee - ginger ale flavor), flavored milk
	Untaxed	Powdered juice (Tang), SSB coffee & tea, powdered milk
**7-.8.9**	Taxed	Malt, Energy drinks (Plus), juice drinks
	Untaxed	Powdered juice (Turbo), SSB coffee & tea, iced tea
**9-10.9**	Taxed	Soda (Sprite, Busta), flavored milk
	Untaxed	Powdered juice (Koolaid), homemade sweet juice, NAS juice
**11.-12.9**	Taxed	Soda (Coca Cola, Frutee), juice drinks (Pinehill Dairy, Fruta)
	Untaxed	Lemonade, NAS juice, homemade juices/shakes/punch
**13-14.9**	Taxed	Juice drinks (Pinehill Dairy, Fruta), soda (Frutee, Ju-C)
	Untaxed	Mauby, homemade sweetened juice, NAS juice (Pinehill Dairy), SSB coffee & tea
**15-16.9**	Taxed	Soda, juice drinks
	Untaxed	SSB coffee & tea
**70+**	Taxed	NA
	Untaxed	Sweetened condensed milk

Alvarado M, *et al. BMJ Open* 2020; 10:e035981. doi: 10.1136/bmjopen-2019-035981

Appendix Text 1: Definition of beverage categories and nutrient composition

We categorized drinks according to the categories summarized in Appendix Table 2.

Nutribase includes nutrient information from the United States Department of Agriculture (USDA) and Canadian food composition databases. For products not included in Nutribase (e.g. local and regional brands of sodas, juices; internationally produced beverages imported from South Africa, Turkey, etc) we assigned nutrient information (sugars in grams and total calories in kilocalories) based on nutrient label data collected from product packages in stores and from websites. When a specific brand and flavour were reported in the dietary recall, we used nutrient information from the corresponding product. When no flavour was reported, we used the mean nutrient values across a range of available flavours. We relied on Nutribase nutrient information for available international brands (e.g. Coke, Sprite, etc). When no brand was reported in the dietary recall, we used the mean nutrient information for that beverage category.

For powdered drinks (powdered juices and hot chocolate) we used packet instructions to estimate reconstituted levels. Most powdered drinks reported in the recalls already include sugar and do not require additional sugar to be added. While people may add additional sugar, this was not included as a prompt in the standard 24-hr dietary recall, so our estimates of sugar intake from powdered drinks may be an underestimate.

For powdered milk we assumed a 1:5 dilution ratio and corrected levels of total calories and sugars accordingly (since the product was previously entered as undiluted powdered milk in Nutribase).

For homemade SSBs, a previous Barbados-based study used the weighed recipe approach to estimate nutrient content for three popular drinks: mauby, ginger beer and lemonade [51]. For other homemade drinks, we used the recipes that participants reported to identify similar products within Nutribase. Participants had been prompted for recipes and we used the ingredients to identify similar products within Nutribase. For homemade SSBs (smoothies and juice drinks), we categorized these as "fruit punch drink "pina colada," "blended smoothie, banana, oats, milk, honey, yogurt," "flavored milks," "pina colada," "blended shake, milkshake vanilla," "mixed berry fruit smoothie," "fruit 'n' yogurt smoothie, strawberry kiwi," "tropical fruit smoothie," "golden apple juice," "lemonade," "juice apple & cherry juice," "island guava drink," "orange flavor drink" "passion fruit juice," "dock, boiled (sorrel)," "mixed fruit juice," or "grape juice" as appropriate.

For homemade non-SSB (no added sugar smoothies and juices), we categorized these as "blended carrot, beet, celery, cucumber, apple juice without sugar," "cranberry juice," "carrot juice," "V8 60% vegetable juice, V-Lite," "aloe vera juice, " "mango juice," "orange juice, unsweetened," "lemon juice, raw," "passion fruit juice, raw," "soy milk," or "mandarin papaya drink" as appropriate.

Mauby is a local bark that is boiled with water and sugar to make a sweet drink (and can also be bought as a ready-made syrup and diluted at home or purchased ready-to-drink).

Sorrel is a flower (similar to hibiscus) that is used to make a sweetened drink. Golden apples are a fruit that are used to make a juice (often sweetened).

We excluded snowcones, as we considered these to be a dessert and not a drink.

Several drinks were categorized within Nutribase as "Pina coladas" although upon review these were identified to be homemade punches or smoothies. The sugar and total calories content of these four observation were rescaled, with pineapple punch and coconut punch re-scaled based on "fruit punch

drink" and "smoothie homemade" and "mango shake, homemade blended almond milk" re-scaled based on ("blended smoothie - banana oats milk honey yogurt").

To exclude galactose and lactose sugars, we subtracted these from total sugars. Where Nutribase did not automatically assign lactose/galactose sugar content to milk products, we assumed all sugars were from lactose/galactose in no added sugar milk products.

When sweetened condensed milk was reported with coffee/tea, we estimated the total sugar concentration per quantity of coffee/tea consumed and reported this under "sweetened tea/coffee" rather than "sweetened condensed milk." When consumption was reported with cereal or cream of wheat in place of regular milk we reported this under "sweetened condensed milk."

Throughout this report, "SSBs" refer to both taxed and untaxed SSBs (excluding non-SSBs), while "soft drinks" refer to both SSBs and non-SSBs. Some non-SSBs (such as no added sugar juice) contain free sugars. To clarify when non-SSBs are included, we refer to "soft-drinks" rather than "SSBs."

For participants with more than two recalls (n=1) we used only the first two recalls, assuming that reporting quality may have changed with repeated exposure to the survey instrument.

We converted all reported beverage volumes into milliliters.

Appendix Text 2: Sugar concentration by product types

The sugar concentration of some product types varied greatly, such as for home-prepared SSB tea/coffee with reported consumption at almost every sugar concentration level. Other product types were more narrowly defined (such as flavoured water, which was only found in the 3-4.9 gr/100mL category). Most of the sweetest products (13+ gr/100mL) were locally or regionally produced fruit drinks or sodas. Some flavours of no added sugar juice (non-SSBs) had a higher sugar concentration than juice drinks (SSBs), and some flavours of sodas had notably lower levels of sugar concentration than other flavours under the same brand. Sweetened condensed milk consumed as a milk substitute with cereal or cream of wheat was the only product with a sugar concentration greater than 17+ gr/100mL.

Appendix Figure 1: Distribution of SSB consumption in mean daily servings/capita, given any SSB consumption, Barbados 2012-2013: the Barbados Salt Intake Study

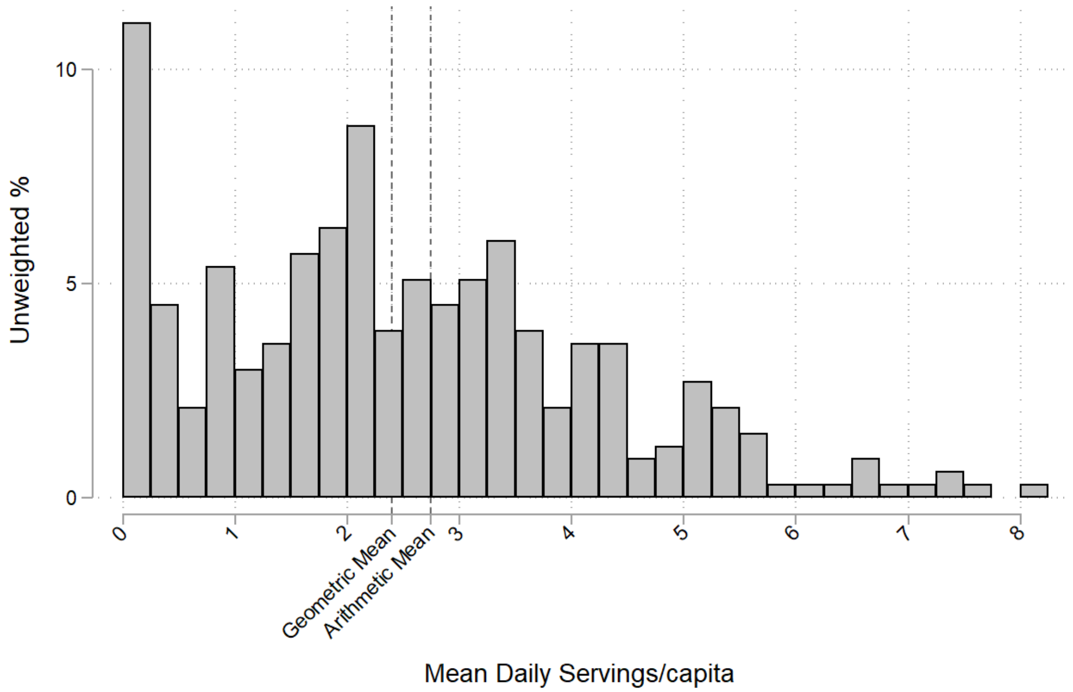

Appendix Figure 2: Per-person mean Total Energy Intake (TEI) attributable to SSB-related free sugars consumption (%), given any consumption, Barbados 2012-2013: the Barbados Salt Intake Study

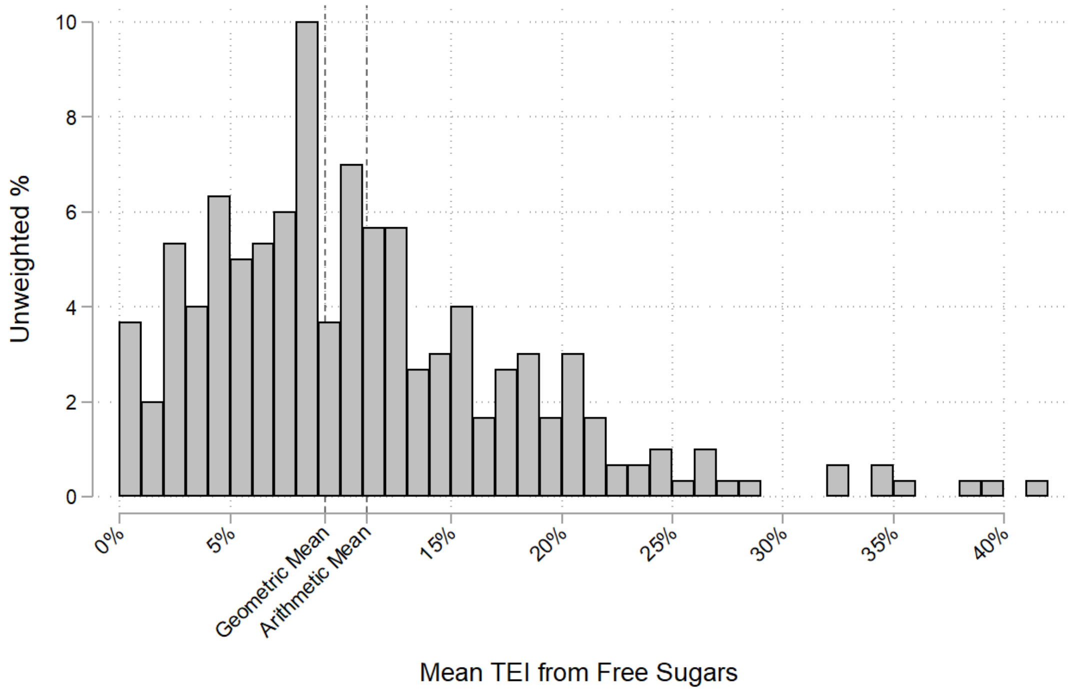

Appendix Figure 3: Mean per-person free sugar consumed from soft drinks amongst adults aged 25-64, by product type and taxable status, Barbados 2012-2013: the Barbados Salt Intake Study

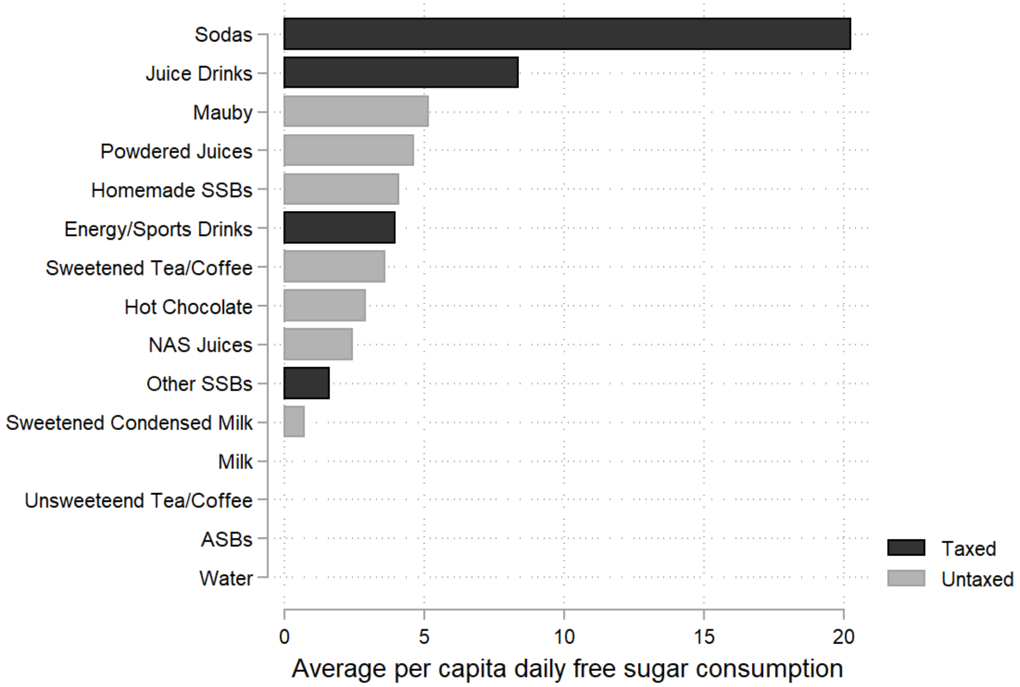

Alvarado M, *et al. BMJ Open* 2020; 10:e035981. doi: 10.1136/bmjopen-2019-035981
