# Supplementary material for: Using nutritional survey data to inform the design of sugar-sweetened beverage taxes in low-resource contexts: a cross-sectional analysis based on data from an adult Caribbean population
Source: BMJ Open. 2020 Sep 10;10(9):e035981. doi: 10.1136/bmjopen-2019-035981 (PMC7485232; doi:10.1136/bmjopen-2019-035981)
Supplement: Author's manuscript [file bmjopen-2019-035981.draft_revisions.pdf]

BMJ Open is committed to open peer review. As part of this commitment we make the peer review history of every article we publish publicly available.

When an article is published we post the peer reviewers' comments and the authors' responses online. We also post the versions of the paper that were used during peer review. These are the versions that the peer review comments apply to.

The versions of the paper that follow are the versions that were submitted during the peer review process. They are not the versions of record or the final published versions. They should not be cited or distributed as the published version of this manuscript.

BMJ Open is an open access journal and the full, final, typeset and author-corrected version of record of the manuscript is available on our site with no access controls, subscription charges or pay-per-view fees (http://bmjopen.bmj.com).

If you have any questions on BMJ Open's open peer review process please email info.bmjopen@bmj.com

BMJ Open

Using nutritional survey data to inform the design of sugar-sweetened beverage taxes in low-resource contexts: a cross-sectional analysis based on data from an adult Caribbean population

Journal:	*BMJ Open*
Manuscript ID	bmjopen-2019-035981
Article Type:	Original research
Date Submitted by the Author:	26-Nov-2019
Complete List of Authors:	Alvarado, Miriam; University of Cambridge, MRC Epidemiology Unit<br>Harris, Rachel; University of the West Indies at Cave Hill, Faculty of Medical Sciences<br>Rose, Angela; University of the West Indies at Cave Hill, George Alleyne Chronic Disease Research Centre, Caribbean Institute for Health Research<br>Unwin, Nigel; University of Cambridge, Global Diet and Activity Research, MRC Epidemiology Unit<br>Hambleton, Ian; University of the West Indies at Cave Hill, George Alleyne Chronic Disease Research Centre, Caribbean Institute for Health Research<br>Imamura, Fumiaki; University of Cambridge, MRC Epidemiology Unit<br>Adams, J; University of Cambridge, Centre for Diet and Activity Research, MRC Epidemiology Unit
Keywords:	Health policy < HEALTH SERVICES ADMINISTRATION & MANAGEMENT, Nutrition < TROPICAL MEDICINE, Epidemiology < TROPICAL MEDICINE, Public health < INFECTIOUS DISEASES

SCHOLARONE™
Manuscripts

I, the Submitting Author has the right to grant and does grant on behalf of all authors of the Work (as defined in the below author licence), an exclusive licence and/or a non-exclusive licence for contributions from authors who are: i) UK Crown employees; ii) where BMJ has agreed a CC-BY licence shall apply, and/or iii) in accordance with the terms applicable for US Federal Government officers or employees acting as part of their official duties; on a worldwide, perpetual, irrevocable, royalty-free basis to BMJ Publishing Group Ltd ("BMJ") its licensees and where the relevant Journal is co-owned by BMJ to the co-owners of the Journal, to publish the Work in this journal and any other BMJ products and to exploit all rights, as set out in our licence.

The Submitting Author accepts and understands that any supply made under these terms is made by BMJ to the Submitting Author unless you are acting as an employee on behalf of your employer or a postgraduate student of an affiliated institution which is paying any applicable article publishing charge ("APC") for Open Access articles. Where the Submitting Author wishes to make the Work available on an Open Access basis (and intends to pay the relevant APC), the terms of reuse of such Open Access shall be governed by a Creative Commons licence – details of these licences and which Creative Commons licence will apply to this Work are set out in our licence referred to above.

Other than as permitted in any relevant BMJ Author's Self Archiving Policies, I confirm this Work has not been accepted for publication elsewhere, is not being considered for publication elsewhere and does not duplicate material already published. I confirm all authors consent to publication of this Work and authorise the granting of this licence.

For peer review only

Title: Using nutritional survey data to inform the design of sugar-sweetened beverage taxes in low-resource contexts: a cross-sectional analysis based on data from an adult Caribbean population

Authors

Miriam Alvarado (corresponding author)

Centre for Diet and Activity Research, MRC Epidemiology Unit, University of Cambridge, Cambridge, United Kingdom

mra47@cam.ac.uk

http://orcid.org/0000-0003-2864-9410

+44 7944524573

Rachel Harris, Faculty of Medical Sciences, The University of the West Indies, Cave Hill, Barbados, rachel.harris@mycavehill.uwi.edu

Orchid identifier:0000-0002-5205-4989

Angela Rose, George Alleyne Chronic Disease Research Centre, Caribbean Institute for Health Research, The University of the West Indies, Bridgetown, Barbados, angela.rose@epi-research.com

https://orcid.org/0000-0002-2493-8082

Nigel Unwin, Global Diet and Activity Research, MRC Epidemiology Unit, University of Cambridge, Cambridge, United Kingdom, nigel.unwin@mrc-epid.cam.ac.uk

https://orcid.org/0000-0002-1368-1648

Ian Hambleton, George Alleyne Chronic Disease Research Centre, Caribbean Institute for Health Research, The University of the West Indies, Bridgetown, Barbados, ian.hambleton@cavehill.uwi.edu;

https://orcid.org/0000-0002-5638-9794

For peer review only - http://bmjopen.bmj.com/site/about/guidelines.xhtml

Fumiaki Imamura, MRC Epidemiology Unit, University of Cambridge, Cambridge, United Kingdom, Fumiaki.Imamura@mrc-epid.cam.ac.uk

https://orcid.org/0000-0002-6841-8396

Jean Adams, Centre for Diet and Activity Research, MRC Epidemiology Unit, University of Cambridge, Cambridge, United Kingdom, jma79@medschl.cam.ac.uk

http://orcid.org/0000-0002-5733-7830

Keywords: Health policy, Nutrition, Epidemiology, Prevention strategies, Public Health

Word count: 3071

Reference count: 49

For peer review only

For peer review only - http://bmjopen.bmj.com/site/about/guidelines.xhtml

Abstract

Objective

Sugar-sweetened beverage (SSB) taxes have been implemented widely. We propose three criteria that can be used to improve SSB tax design with the goal of reducing free sugar consumption: 1) high baseline consumption of SSBs and SSB-derived free sugars, 2) high percentage of SSB-derived free sugars covered by the tax, and 3) consistent differentiation between high- and low-sugar SSBs. We aimed to evaluate these criteria using pre-existing nutritional survey data in a developing economy setting.

Methods

We used data from a nationally representative cross-sectional survey in Barbados (2012-2013, prior to SSB tax implementation). Data were available on 334 adults (25-64 years) who completed two non-consecutive 24-hour dietary recalls. We estimated the prevalence of SSB consumption and its contribution to total energy intake, overall and stratified by taxable status. We assessed the percentage of SSB-derived free sugars subject to the tax and identified the consumption-weighted sugar concentration of SSBs, stratified by taxable status.

Findings

Accounting for sampling probability, 88.8% of adults (95%CI 85.1,92.5) reported SSB consumption, with a geometric mean of 2.4 servings/day (±2×standard deviation, 0.6,9.2) among SSB consumers. Sixty percent (95%CI 54.6, 65.4) of SSB-derived free sugars would be subject to the tax. The tax did not clearly differentiate between high- and low-sugar beverages.

Conclusion

Given high SSB consumption, targeting SSBs was a sensible strategy in this setting. A substantial percentage of free sugars from SSBs were not covered by the tax, reducing possible health benefits. The criteria proposed here may help policymakers to design more effective SSB taxes.

For peer review only - http://bmjopen.bmj.com/site/about/guidelines.xhtml

Strengths and limitations of this study

- A nationally representative dietary survey with two non-consecutive 24-hour dietary recalls allowed assessment of sugar-sweetened beverage (SSB) consumption patterns prior to the introduction of a tax on SSBs.
- Twenty-four-hour dietary recalls may be subject to reporting bias and may underestimate total SSB intake.
- Energy density (% of total energy intake) is reported to partially mitigate potential reporting biases.
- Data were not available on children, adolescents or adults over the age of 65.
- This is the first study that we are aware of to quantify the percentage of SSB-derived free sugars covered by a real-world SSB tax.

For peer review only - http://bmjopen.bmj.com/site/about/guidelines.xhtml

Background & Rationale

Background

The World Health Organization (WHO) has recommended limiting free sugar consumption to less than 10% of total energy intake (TEI)[1]. Free sugars include sugars added to food and beverages, as well as sugars in fruit juices[1]. Sugar-sweetened beverages (SSBs) are a major source of free sugars, and consumption of SSBs is associated with higher risk of diabetes, certain cancers and obesity[2–10].

Given these health risks, the WHO and others have recommended taxing SSBs to reduce consumption[11–15]. A number of countries (including many small island developing states (SIDS) and low- and middle-income countries) have introduced SSB taxes, at least in part for health reasons[12,16–19]. However, these taxes vary widely in design[16]. In some settings, taxable products have been narrowly defined, whereas elsewhere they have been defined to include all soft drinks (even those containing no or small amounts of free sugars)[16,20]. These differences are likely to have important health implications[21].

We propose three criteria, drawing on current guidance, that can be used to improve the design of SSB taxes with the goal of reducing free sugar consumption[12,21,22].

First, SSB taxes are more likely to be effective in places where SSB consumption levels are high and where SSB-derived free sugars represent a high proportion of total energy intake[23]. As Singh et al. have demonstrated, there is great heterogeneity in SSB consumption levels worldwide[24]. In terms of reducing current free sugar consumption, SSB taxes have the greatest potential in settings with high baseline consumption.

Second, SSB taxes should cover a high proportion of regularly consumed SSBs, reducing substitution incentives[12]. If taxes are applied on a limited proportion of total SSBs consumed in a given population,

For peer review only - http://bmjopen.bmj.com/site/about/guidelines.xhtml

the potential impact on health will be necessarily limited. If consumers substitute towards untaxed SSBs, health goals will be further undermined.

Finally, SSB taxes should consistently differentiate between high- and low-sugar products[25,26]. If SSB taxes are not consistently applied on all high-sugar SSBs, health goals will be further undermined especially if consumers substitute towards high-sugar untaxed SSBs. Box 1 summarizes these criteria.

The assessment of these criteria should be informed by local consumption patterns as much as possible. Commercial purchase data (such as Nielsen and Kantar consumer panels) have been used to assess SSB consumption patterns in the US and the UK, but these data are costly and unavailable in some settings[26,27]. In lower-resource settings in particular, it may be pragmatic to use pre-existing nutritional survey data to help inform context-specific policy design[28,29]. A recent review demonstrated that individual level dietary surveys have been conducted in at least 116 countries, representing 88.7% of the global 2010 adult population [28,29]. These nutritional survey data may provide a feasible way to evaluate these proposed criteria more widely.

Case Study: The Barbados SSB Tax

The Government of Barbados implemented a 10% SSB tax in 2015[18]. Taxable products (both imported and locally manufactured) were defined according to the Harmonized System (HS) tariff classifications and included soda, juice drinks, energy and sports drinks [18,30]. Some SSBs were not included in the tax definition, such as sugar-sweetened drink mixes (e.g. powdered juice and powdered hot chocolate) and sugar-sweetened syrups (e.g. mauby[1] syrups)[18].

A nationally representative nutritional survey was conducted in 2012-2013, well in advance of the introduction of the Barbados SSB tax in 2015. We revisited these data to assess the tax according to our

[1] Mauby is a local drink, which is typically sold either as a syrup to be reconstituted at home or as dried bark for home preparation

For peer review only - http://bmjopen.bmj.com/site/about/guidelines.xhtml

proposed criteria. We established three research questions: 1) what were pre-tax SSB consumption levels (in terms of volume and contribution to TEI)? 2) what percentage of SSB-related free sugars were covered by the tax? And 3) did the tax clearly differentiate between low and high-sugar beverages? We aimed to assess whether it was feasible to evaluate these criteria in a low-resource setting using existing nutritional survey data.

Methods

Study Design & Population

For peer review only

We used nutritional survey data from Barbados, a country with a population of 293,131 (2018 estimate) and $18,600 GDP/capita (2017 estimate) [31]. Barbados is likely to share characteristics with low/middle-income and other SIDS settings (limited access to commercial sales data, a product-based definition of taxable products, etc.).

The data used in this study were from the Health of the Nation study, conducted between June 2012 and November 2013 (with a response rate of 54% and final sample size of 1,234). Details of the overall sampling design, study recruitment and study procedures have been summarized elsewhere[32]. A sub-sample of 441 participants aged 25 to 64 were randomly selected to complete two non-consecutive in-person 24-hour dietary recalls [33]. Three hundred and sixty-eight participants (83%) consented to participate (for a combined response rate of 45%).

Each dietary recall was collected at home by a trained interviewer, using a standard multi-pass probing method, three-dimensional standardized food models and familiar measuring units[34]. Recalls were evenly distributed across quarters, with the exception of July-September when fewer recalls were conducted. The average time between the first and second recall was six days, and recalls were evenly distributed by day of the week. Data were processed using Nutribase Pro software[35]. Survey weights were used to reflect the clustered sampling design, to take into account the combined non-response rate

For peer review only - http://bmjopen.bmj.com/site/about/guidelines.xhtml

and to match the age and sex distribution of the Barbados population as captured in the Barbados 2010 Census[33].

We excluded participants with reported caloric intake less than 500 kcal/day or greater than 5000 kcal/day (n=5), those with missing covariate data (n=21), those with only one recall (n=1), and those with missing survey weights (n=7), leaving a total of 334 participants.

Ethics approval was given by the University of the West Indies Cavehill Institutional Review Board.

Patient and public involvement

Participants were not involved in the design, conduct, reporting or dissemination of these analyses.

Measures of SSB Consumption

We estimated the prevalence of SSB consumption, defined as those with any reported SSB consumption on at least one day. Next, we estimated average volume consumed (mean SSB servings/day) amongst SSB consumers (excluding those who did not report any SSB consumption). A serving was defined as 250 mL [6]. We reviewed each dietary recall and extracted product information for all reported SSBs.

Soft drinks were categorized based on whether they contained added sugars and whether they were subject to the Barbados SSB tax. Taxed SSBs included soda, juice drinks, energy/sports/malt drinks and other taxed SSBs; untaxed SSBs included sugar-sweetened powders (powdered juice drinks, hot chocolate), sugar-sweetened syrups (mauby), sweetened tea/coffee, sweetened condensed milk and other untaxed SSBs; and untaxed non-SSBs included water, no added sugar (NAS) fruit juice, milk, entirely artificially-sweetened beverages (ASBs) and other non-SSBs (see Appendix Table 1).

We identified nutrient content for every beverage at the most detailed level possible (e.g. brand, flavour). We relied on Nutribase nutrient content for international brands (and cross-checked these with local nutrient information panels for consistency). For brands not included in Nutribase, we collected nutrient information directly from product packaging and manufacturer websites (see Appendix Text 1).

For peer review only - http://bmjopen.bmj.com/site/about/guidelines.xhtml

Covariates

Demographic information and education history were collected at the first visit. We dichotomized age (25-44 years old, 45-64 years old) and education (secondary education or less compared to tertiary education, which included undergraduate, postgraduate and technical/vocational training).

Statistical Methods

1: Levels of SSB consumption

We estimated the prevalence of SSB consumption, and descriptive statistics (mean ± 1.96×standard deviation) of levels of SSB consumption among consumers and the percentage of TEI from SSB-derived free sugars, stratified by covariates. Since SSB consumption was right-skewed (see Appendix Figures 1 and 2), we report volume and percent of TEI using geometric means and SD. To enable comparison with global estimates, we re-estimated overall SSB intake only using the arithmetic mean, including non-consumers and using 8 oz. as a serving size.[2]

2: Percentage of SSB-derived free sugars captured by tax

We re-estimated the prevalence of SSB consumption and percentage of TEI attributable to SSB-derived free sugars separately for taxed and untaxed SSBs. Then we calculated the percentage of total SSB-derived free sugars[3] subject to the tax.

3: Free-sugar concentration

We estimated mean free-sugar concentration by SSB sub-category (i.e. separately for sodas, SSB juice drinks, etc.), weighted by reported consumption.[4] To illustrate how nutritional survey data may be used to

---

[2] Defined by Singh et al. as 226.8 grams (equivalent to 226.8 mL), or 8 imperial ounces[24]
[3] In calculating total SSB-derived free sugars, we excluded free sugars from non-SSBs (such as free sugars in NAS juice) and sugars naturally present in milk (which are not included in the definition of free sugars)[1].

[4] Consumption-weighted estimates of free-sugar concentration were used to reflect consumption patterns (compared to reflecting the distribution of available free-sugars in the market)

For peer review only - http://bmjopen.bmj.com/site/about/guidelines.xhtml

assess potential SSB tax tiers, we report mean per-person daily volume consumed by grams of free sugar per 100mL.

All analyses were weighted by sampling probability and conducted using Stata 14.0 (StataCorp LP, Texas, United States).

This study is reported according to the Strengthening Reporting of Observational Studies in Epidemiology Extension for Nutritional Epidemiology (STROBE-nut) checklist (see Appendix Table 2) [36].

Results

1: Levels of SSB consumption

Eighty-eight percent of participants reported consuming SSBs at least once over the two days (Table 1). Prevalence of SSB consumption did not differ significantly between sub-groups. Amongst those who reported any consumption, mean per-person daily SSB intake was 2.4 250mL servings (mean±2 SD, 0.6,9.2). To enable comparison with published estimates, we also report mean per-person daily SSB intake in 8 oz. servings across the whole study population (2.7 8 oz. servings (95%CI 2.5, 2.9)). Men and those with less education reported consuming a higher volume of SSBs than their counterparts (p-values of <0.001 and 0.004 respectively). TEI from SSB-related free sugars was 9.2% (mean±2×SD, 2.1,41.3), with a similar patterning of results by sub-groups.

2: Percentage captured by tax

Seventy five percent of participants consumed taxed SSBs, and a similar percentage consumed untaxed SSBs (Table 2). A higher percentage of men consumed taxed SSBs as compared to women (p=0.035). TEI attributable to taxed SSBs was 6.7% (mean±2SD 1.7,26.5), and TEI attributable to untaxed SSBs was 3.5% (mean±2SD 0.4,27.3). Those with less education consumed a higher percentage of TEI from taxed

For peer review only - http://bmjopen.bmj.com/site/about/guidelines.xhtml

SSBs than those with higher education (p=0.01). Sixty-one percent of SSB-derived free sugars were taxed (95%CI 55.7,66.5), with no significant differences by sub-group.

3: Free-sugar concentration

We estimated mean consumption-weighted free sugar concentration for each product category. As summarized in Figure 1, sweetened condensed milk was associated with the highest concentration of free sugars (70 gr/100mL). Mauby, juice drinks, and sodas had the next highest average free sugar concentrations. Five of the nine beverage types with more than 6.25 grams free sugar/100 mL (Chile's SSB tax threshold) were untaxed. We also report mean per-person free sugar consumed (taking into account sugar concentration and consumption levels), by product type (see Appendix Figure 3). We assessed the mean per-person daily consumption of soft drinks (excluding those with free sugar<1 gr/100mL, and including home-prepared SSBs and no added sugar juice) by free sugar concentration (Figure 2), stratified by taxed/untaxed SSBs. Half of the drinks consumed with the highest free sugar levels (12+gr/100mL) were not subject to the tax (see Appendix Table 3 and Appendix Text 2 for examples of the products in each category by free sugar concentration).

Discussion

We proposed three criteria for evaluating the design of SSB taxes and demonstrated that pre-existing nutritional survey data may be used to address these criteria with important implications for tax design.

SSB consumption levels amongst adults aged 25-64 years in Barbados were very high (2.7 8-oz. servings/day, 95% CI 2.5,2.9) compared to global estimates (0.58 8-oz. servings/day, 95%CI 0.37, 0.83)[24]. SSB-derived free sugar accounted for 9.2% of TEI (mean±2 SD 2.1,41.3), and therefore nearly half of the population exceeded the WHO's recommendation for *total* free sugar (10%, including sweets, jams, confectionary, etc.) solely from SSB consumption[1].

For peer review only - http://bmjopen.bmj.com/site/about/guidelines.xhtml

The Barbados SSB tax captured a moderate percentage of SSB-derived free sugars (61.1%, 95% CI 55.7,66.5), possibly incentivizing substitution to untaxed SSBs and dampening the potential health impact of the tax.

The Barbados SSB tax did not clearly differentiate between consumption-weighted high- and low-sugar products, which may further incentivize substitution to high-sugar untaxed alternatives in particular.

Strengths and Limitations

The proposed criteria reflect some aspects of SSB tax design, but additional context-specific factors need to be considered (e.g. public acceptability, market structure, etc.). However, applying these criteria illustrated important aspects of context-specific consumption patterns and may provide useful information to policymakers.

Given the data available, we were not able to assess SSB consumption patterns amongst children, young adults or adults over 65. The combined response rate was 45%, (comparable to that of a similar national dietary survey in the United Kingdom (47%)[37]), and survey weights were used to take the population representativeness into account as much as possible and to match the age and sex distribution of the Barbados population. There was a dip in recalls conducted between July-September, suggesting that recall data may be slightly seasonally biased. July-September represent the hottest months in Barbados and SSB consumption may increase during these months, which would imply that our estimate may have been underestimated[38]. Underestimation could have also occurred because of the subjectivity in the two 24-hour recall data, which may have been partially mitigated by the energy density approach (% of TEI).

In relation to other studies

The Global Burden of Disease (GBD) 2010 study estimated that SSB consumption in Barbados was between 2.0-2.4 8-oz servings/day, higher than our comparable estimate of 2.7 servings/day [24]. This difference may reflect that the GBD estimate for Barbados was derived from a study conducted in Jamaica between 1993-1995[39] and an unpublished analysis[24].

For peer review only - http://bmjopen.bmj.com/site/about/guidelines.xhtml

In comparison to national measures of SSB consumption from other settings, our estimates were relatively high (criterion 1). Han & Powell estimated the two-day prevalence of SSB consumption amongst US adults was 50%, lower than our comparable estimate of 89% amongst adults in Barbados[40]. A study of Dutch adults found that SSBs and non-SSBs accounted for 5.1% of TEI and a study of Australian children estimated an SSB contribution of 4.4%, much lower than our 9.2% estimate[2,41,42].

This is the first study that we are aware of to quantify the percentage of SSB-derived free sugars covered by an SSB tax (criteria 2,3). Given heterogeneous SSB consumption worldwide, it would be valuable to repeat this approach in different settings to assess both the potential (in general) of an SSB tax to target sources of soft drink-derived free sugar, as well as to evaluate the specific definition of proposed future taxes. Powell et al. have assessed the distribution of sugar concentration by consumption of ready-to-drink SSBs (excluding home-prepared SSBs) in the US, and identified three clusters of highly-consumed concentration levels[26]. They recommended that SSB tax thresholds should be set at 5 gr/8 oz. below these highly-consumed clusters to encourage reformulation[26]. This guidance would imply a threshold of around 8gr/100mL given the distribution we observed in Barbados, somewhat higher than the threshold used in Chile (6.25gr/100mL)[43]. More empirical work is needed to understand how companies respond to these thresholds in practice, and to assess how home-prepared SSBs compare in terms of sugar concentration levels in other settings.

Meaning of the study

Implications for Barbados

Adult SSB consumption levels were high before the introduction of the Barbados SSB tax. However, the definition of taxable products suggests that the tax was only likely to cover a moderate proportion of SSB-related free sugar consumption. While the Barbados tax was amended in 2017 to include store-bought mauby syrup, homemade mauby and other homemade SSBs remain difficult to address through a

For peer review only - http://bmjopen.bmj.com/site/about/guidelines.xhtml

tax[44]. To maximize health benefit, the tax could be further amended to cover a higher proportion of SSB-derived free sugars, such as powdered juice drinks and powdered hot chocolate.

Some untaxed products (e.g. no added sugar juices, powdered juices) contain higher levels of free sugars than taxed products, suggesting that substitution to untaxed beverages could have the unintended consequence of increasing sugar consumption. Recent dietary guidelines in Barbados suggest limiting juice intake to 250 ml/day, and similar guidelines in the UK recommend a threshold of less than 150ml/day. Some consideration to including no added sugar juices in the SSB tax may further help to deter free sugar consumption[45–47].

Implications for other settings

We found that the proposed criteria were simple to assess and generated useful insight, given existing best practice recommendations for developing SSB taxes[12].

Taxable products defined by product category (i.e. definitions based on tariff codes, as has been adopted in other SIDS and lower-resource settings such as St. Kitts and Nevis, Bolivia and South Africa[48–50]) may vary widely. When SSB taxes are defined by product categories, care should be taken that all high-sugar products are taxed to limit incentives for substitution.

A potential limitation of SSB taxes in general is that they do not cover home-prepared SSBs. In contexts where a high absolute volume of SSBs are home-prepared, an SSB tax has less health potential irrespective of the definition of taxable products. Complementary mass media or education campaigns that target untaxed sources of SSB-derived free sugars may be helpful in addressing free sugar consumption overall, given the limitations of any tax to capture all of these beverages.

It was feasible to assess our proposed criteria using existing nutritional survey data, and these data offered some advantages over other potential data sources. Nutritional survey data can provide insight around homemade and on-the-go SSB consumption, although they may be limited by small sample sizes (which may preclude sub-group analyses) and infrequent administration. Nevertheless, standard nutritional

For peer review only - http://bmjopen.bmj.com/site/about/guidelines.xhtml

surveys, when combined with detailed nutrient content data, can provide an opportunity to assess consumption patterns and highlight opportunities to design tailored, context-informed SSB taxes.

Conclusion

We used nutritional survey data to demonstrate high levels of SSB consumption (both in volume and as a percentage of total energy intake) amongst adults in Barbados prior to the introduction of the Barbados SSB tax. The Barbados SSB tax could be amended to apply to additional SSB products, potentially increasing possible health benefits. SSB taxes may miss home-prepared SSBs, and additional interventions may be needed to address these sources of free-sugars. Evaluating the criteria we propose here (baseline SSB consumption levels, the percentage of all SSBs that would be taxed, and the ability of a tax to differentiate between high- and low-sugar soft drinks) in other settings may help to improve SSB tax design and increase potential positive health impacts.

For peer review only - http://bmjopen.bmj.com/site/about/guidelines.xhtml

For peer review only

Box 1: Proposed criteria to help inform design of sugar-sweetened beverage (SSB) taxes from a health perspective

Box 1: Proposed criteria to help inform design of sugar-sweetened beverage (SSB) taxes from a health perspective

1. High baseline levels of SSB consumption and high contribution of SSB-derived free sugar to total energy intake (TEI)
2. High percentage of SSB consumption covered by SSB tax
3. Clear distinction made by SSB tax between high- and low-sugar SSBs

Table 1: Consumption of sugar-sweetened beverages amongst adults aged 25-64 years by demographic characteristics, Barbados 2012-2013: Barbados Salt Intake Study (n=334)

		**Distribution** (n=334)	**Prevalence of any SSB consumption**[1] (n=334)		**Volume (servings/day), given SSB consumption**[2,3] (n=300)		**TEI from SSB free sugars**, given SSB consumption**[2,5] (n=300)	
		%	%	95% CI	Mean	Mean±2 SD[4]	%	Mean±2 SD[4]
Overall	Total		88.8	85.1,92.5	2.4	0.6,9.2	9.2	2.1,41.3
By Subgroup								
Age	*25-44*	51.1	89.1	83.7,94.6	2.7	0.9,8.3	10.3	3.0,35.6
	45-64	48.9	88.4	82.1,94.7	2.2	0.5,9.8	8.2	1.5,46.6
Sex	*Males*	48.8	89.7	83.7,95.7	2.8*	0.9,9.1	10.5*	2.7,41.3
	Females	51.2	87.9	83.0,92.9	2.1*	0.5,8.6	8.2*	1.7,39.6
Education	*<Tertiary*	62.9	90.9	85.7,96.2	2.7*	0.8,9.2	10.0*	2.4,41.3
	Tertiary+	37.1	85.1	78.1,92.2	2.0*	0.5,8.4	8.0*	1.6,39.6

* Significant at p-value<0.05 in survey-weighted bivariate logistic regression (prevalence of any SSB consumption models) or survey-weighted bivariate generalized linear regression with log-link function (volume, TEI models)
[1] Defined as >0 gr of any SSB across two 24-hour recalls
[2] Geometric means
[3] Defined as the mean volume (250 mL servings/day) from SSBs, amongst all SSB-consumers. For estimates of 8 oz per serving, each value is to be multiplied by 0.91.
[4] Geometric mean±1.96 SD derived from the log-transformed variable to reflect the sample distribution
[5] Defined as the percentage of total energy intake (TEI) from SSB-derived free sugars, amongst all SSB-consumers

For peer review only - http://bmjopen.bmj.com/site/about/guidelines.xhtml

Table 2: Prevalence of consumption and total energy intake (TEI) (%) from sugar-sweetened beverage (SSB)-derived free sugars among adults aged 25 to 64 years, stratified by subsequent taxable status, Barbados 2012-2013: Barbados Salt Intake Study (n=334)[1]

		**Prevalence of any SSB Consumption[2]**				**TEI** from SSB-derived free sugars, given any SSB consumption[3,4]**				**SSB-derived free sugars from taxed SSBs[6]**	
		Taxed SSBs (n=334)		Untaxed SSBs (n=334)		Taxed SSBs (n=239)		Untaxed SSBs (n=249)		Percentage Taxed (n=300)	
		%	95% CI	%	95% CI	%	Mean±2 SD	%	Mean±2 SD	%	95% CI
Overall	Total	74.6	69.8,79.5	74.5	69.8,79.2	6.7	1.7,26.5	3.5	0.4,27.3	61.1	55.7,66.5
By Subgroup											
Age	*25-44*	80.8	73.7,87.9	75.0	67.5,82.6	7.0	1.9,25.7	3.6	0.6,22.6	64.2	58.3,70.1
	45-64	68.1	59.5,76.8	74.0	65.5,82.4	6.3	1.4,27.3	3.4	0.3,33.3	57.0	47.5,66.5
Sex	*Males*	79.8*	72.8,86.8	70.8	62.3,79.3	7.2	1.9,27.9	3.9	0.5,30.4	62.1	56.0,68.2
	Females	69.7*	63.1,76.3	78.0	72.9,83.2	6.1	1.5,24.6	3.2	0.4,24.1	59.6	51.5,67.7
Education	*<tertiary*	77.4	70.5,84.3	76.8	70.2,83.5	7.2*	1.8,28.6	3.5	0.5,26.9	63.2	57.7,68.7
	Tertiary+	69.9	61.8,78.0	70.6	63.5,77.7	5.7*	1.5,21.8	3.4	0.4,28.0	56.7	48.1,65.3

* Significant at p-value<0.05 in survey-weighted bivariate logistic regression (prevalence of any SSB consumption models) or bivariate generalized linear regression with log-link function (TEI)

[1] The tax was introduced in 2015, so we retrospectively apply the definition of taxable goods to consumption data reported from 2012-2013
[2]Defined as >0 gr of taxed/untaxed SSBs across two 24-hour recalls
[3] Geometric means
[4] Defined as the mean total energy intake (TEI) from SSB-derived free sugars divided by TEI, amongst all taxed and untaxed SSB-consumers separately
[5] 95% confidence interval defined as mean±1.96 SD to reflect the sample distribution
[6] Defined as the percentage of SSB-derived free sugars that were included in the original Barbados SSB tax definition of taxable products, amongst all SSB-consumers

For peer review only - http://bmjopen.bmj.com/site/about/guidelines.xhtml

Figure_1_file

[1] We present no added sugar (NAS) juice sugars for comparison in the figure, and include a dashed line to represent the SSB tax threshold used in Chile (6.25 gr sugar/100mL) [1,43].
[2] The tax was introduced in 2015, so we retrospectively apply the definition of taxable goods to consumption data reported from 2012-2013

For peer review only

For peer review only - http://bmjopen.bmj.com/site/about/guidelines.xhtml

Figure_2_file

[1] The tax was introduced in 2015, so we retrospectively apply the definition of taxable goods to consumption data reported from 2012-2013

For peer review only

For peer review only - http://bmjopen.bmj.com/site/about/guidelines.xhtml

Acknowledgements

The full Barbados SSB Tax Evaluation Group is: Trevor Hassell (Chairperson of the group, National NCD Commission of Barbados and Healthy Caribbean Coalition); Miriam Alvarado (CEDAR, University of Cambridge); Kenneth George (Ministry of Health), Ian Hambleton (UWI); Maisha Hutton (Healthy Caribbean Coalition); Winston Moore (UWI); Madhuvanti Murphy (UWI); Alafia Samuels (UWI); Godfrey Xuereb (PAHO); Nigel Unwin (UWI; CEDAR, University of Cambridge); Cyril Gill (Ministry of Finance) and Arthur Phillips (Ministry of Health).

The group is supported by a Technical Advisory Committee: Jean Adams (CEDAR, University of Cambridge); Deliana Kostova (US Center for Disease Control and Prevention); Marc Suhrcke (University of York); Anne Marie Thow (University of Sydney)

Nita Forouhi generously offered her time to review a preliminary statistical analysis plan and Dave Collins provided technical expertise around survey-based estimates of SSB consumption.

Funding

The overall evaluation received support from the Canadian International Development Research Centre (grant no. 107604-001) and from the US Center for Disease Control and Prevention (TEPHINET). MA is funded through a Gates Cambridge PhD Scholarship, and received travel funding from Robinson College, the Global Food Security Fund, the Luca D'Agliano Scholarship, the Yates Unilever Fund and the Smuts Memorial Fund. JA is funded by the Centre for Diet and Activity Research (CEDAR), a UKCRC Public Health Research Centre of Excellence. FI was funded by the Medical Research Council Epidemiology Unit Core Support (MC_UU_12015/5). Funding from the British Heart Foundation, Cancer Research UK, Economic and Social Research Council, Medical Research Council, the National Institute for Health Research, and the Wellcome Trust, under the auspices of the UK Clinical Research Collaboration, is gratefully acknowledged. The Health of the Nation Study and the Barbados Salt Intake Study received funding from The Ministry of Health of the Government of Barbados.

The findings and conclusions in this report are those of the authors and do not necessarily represent the official position of any funding agencies.

Conflict of Interest Statement

The authors declare there is no financial conflict of interest.

Ethics Statement

Ethics approval was given by the University of the West Indies Cavehill Institutional Review Board.

Data sharing statement

The datasets generated during and/or analysed during the current study are available from the corresponding author on reasonable request.

For peer review only

Contributors

MA was involved in conceptualizing the study, developing the methodology, analyzing the data and writing and revising the manuscript. NU was involved in conceptualization, funding acquisition, developing the methodology, and reviewing and editing the manuscript. JA was involved in conceptualization, developing the methodology, and reviewing and editing the manuscript. RH was involved in data curation and data quality checks, developing the methodology, and reviewing and editing the manuscript. AH was involved in data curation and software development, developing the methodology, validating results and reviewing and editing the manuscript. IH was involved in data curation, developing the methodology, reviewing data visualizations, and reviewing and editing the manuscript. FI was involved in developing the methodology, reviewing data presentation, and reviewing and editing the manuscript. All authors read and approved the final manuscript.

For peer review only

For peer review only - http://bmjopen.bmj.com/site/about/guidelines.xhtml

References

[1] World Health Organization. Guideline: sugars intake for adults and children. Geneva: WHO UK distributor:Stationery Office; 2015.

[2] Sluik D, van Lee L, Engelen A, et al. Total, Free, and Added Sugar Consumption and Adherence to Guidelines: The Dutch National Food Consumption Survey 2007–2010. Nutrients. 2016;8:70.

[3] Keast DR, Fulgoni VL, Nicklas TA, et al. Food Sources of Energy and Nutrients among Children in the United States: National Health and Nutrition Examination Survey 2003–2006. Nutrients. 2013;5:283–301.

[4] O'Neil CE, Keast DR, Fulgoni VL, et al. Food Sources of Energy and Nutrients among Adults in the US: NHANES 2003–2006. Nutrients. 2012;4:2097–2120.

[5] O'Connor L, Imamura F, Lentjes MAH, et al. Prospective associations and population impact of sweet beverage intake and type 2 diabetes, and effects of substitutions with alternative beverages. Diabetologia. 2015;58:1474–1483.

[6] Imamura F, O'Connor L, Ye Z, et al. Consumption of sugar sweetened beverages, artificially sweetened beverages, and fruit juice and incidence of type 2 diabetes: systematic review, meta-analysis, and estimation of population attributable fraction. BMJ. 2015;351:h3576.

[7] Hu FB. Resolved: There is sufficient scientific evidence that decreasing sugar-sweetened beverage consumption will reduce the prevalence of obesity and obesity-related diseases. Obes. Rev. Off. J. Int. Assoc. Study Obes. 2013;14:606–619.

[8] Chazelas E, Srour B, Desmetz E, et al. Sugary drink consumption and risk of cancer: results from NutriNet-Santé prospective cohort. BMJ. 2019;366:l2408.

[9] Fuchs MA, Sato K, Niedzwiecki D, et al. Sugar-Sweetened Beverage Intake and Cancer Recurrence and Survival in CALGB 89803 (Alliance). PLOS ONE. 2014;9:e99816.

[10] Miles FL, Neuhouser ML, Zhang Z-F. Concentrated sugars and incidence of prostate cancer in a prospective cohort. Br. J. Nutr. 2018;120:703–710.

[11] World Health Organization. Tackling NCDs: "Best buys" and other recommended interventions for the prevention and control of noncommunicable diseases [Internet]. World Health Organization; 2017 [cited 2019 Sep 12]. Available from: https://apps.who.int/iris/bitstream/handle/10665/259232/WHO-NMH-NVI-17.9-eng.pdf?sequence=1&isAllowed=y.

[12] Waqanivalu T, Nederveen L, World Health Organization. Fiscal policies for diet and prevention of noncommunicable diseases: technical meeting report, 5-6 May 2015, Geneva, Switzerland. [Internet]. 2016 [cited 2017 Apr 20]. Available from: http://apps.who.int/iris/bitstream/10665/250131/1/9789241511247-eng.pdf.

[13] World Health Organization. Updated Appendix 3 of the WHO Global NCD Action Plan 2013-2020 [Internet]. 2017 [cited 2019 Oct 9]. Available from: https://www.who.int/ncds/governance/technical_annex.pdf.

For peer review only - http://bmjopen.bmj.com/site/about/guidelines.xhtml

[14] The Task Force on Fiscal Policy for Health. Health-Taxes-to-Save-Lives.pdf [Internet]. 2019 [cited 2019 Jun 9]. Available from: https://www.bbhub.io/dotorg/sites/2/2019/04/Health-Taxes-to-Save-Lives.pdf.

[15] World Health Organization. Taxes on sugary drinks: Why do it? [Internet]. World Health Organization; 2016 [cited 2017 May 17]. Available from: http://apps.who.int/iris/bitstream/10665/250303/1/WHO-NMH-PND-16.5-eng.pdf.

[16] Smith E, Scarborough P, Rayner M, et al. Should we tax unhealthy food and drink? Proc. Nutr. Soc. 2018;77:314–320.

[17] Advocating for Sugar-Sweetened Beverage Tax [Internet]. Johns Hopkins Bloom. Sch. Public Health. [cited 2015 Oct 20]. Available from: http://www.jhsph.edu/departments/health-behavior-and-society/public-health-practice/practice-highlights/advocating-for-sugar-sweetened-beverage-tax.html.

[18] Sinckler C. Presentation of the Financial Statement and Budgetary Proposals 2015 [Internet]. 2015 [cited 2017 May 25]. Available from: https://www.barbadosparliament.com/uploads/document/d1efb84aac6a7abe4c6c0efcf8ceedd2.pdf.

[19] Thow AM, Quested C, Juventin L, et al. Taxing soft drinks in the Pacific: implementation lessons for improving health. Health Promot. Int. 2011;26:55–64.

[20] World Cancer Research Fund International. NOURISHING: Use economic tools to address food affordability and purchase incentives [Internet]. [cited 2018 Nov 9]. Available from: https://www.wcrf.org/sites/default/files/Use-economic-tools.pdf.

[21] Chaloupka FJ, Powell LM, Warner KE. The Use of Excise Taxes to Reduce Tobacco, Alcohol, and Sugary Beverage Consumption. Annu. Rev. Public Health. 2019;40:null.

[22] World Cancer Research Fund International l. Building momentum: lessons on implementing a robust sugar sweetened beverage tax. 2018; Available from: www.wcrf.org/buildingmomentum.

[23] Jou J, Techakehakij W. International application of sugar-sweetened beverage (SSB) taxation in obesity reduction: Factors that may influence policy effectiveness in country-specific contexts. Health Policy. 2012;107:83–90.

[24] Singh GM, Micha R, Khatibzadeh S, et al. Global, Regional, and National Consumption of Sugar-Sweetened Beverages, Fruit Juices, and Milk: A Systematic Assessment of Beverage Intake in 187 Countries. PLOS ONE. 2015;10:e0124845.

[25] Adam AS, Smed S. The effects of different types of taxes on soft-drink consumption. Inst. Food Resour. Econ. 2012;43.

[26] Powell LM, Andreyeva T, Isgor Z. Distribution of Sugar Content in Sugary Drink Purchases in the U.S.: Implications for Tiered Taxation. Available from: https://www.heart.org/-/media/files/about-us/policy-research/policy-positions/sugary-beverages/distribution-of-sugar-content-in-sugary-drink-purchases-in-the-us.pdf?la=en&hash=1780DCF99DD13D6380B36140A403CEEDDDAEC1FA.

For peer review only - http://bmjopen.bmj.com/site/about/guidelines.xhtml

[27] Tedstone A, Targett V, Allen R, et al. Sugar Reduction: The evidence for action [Internet]. London, UK: Public Health England; 2015 [cited 2018 Dec 17]. Available from: https://assets.publishing.service.gov.uk/government/uploads/system/uploads/attachment_data/file/470179/Sugar_reduction_The_evidence_for_action.pdf.

[28] Khatibzadeh S, Saheb Kashaf M, Micha R, et al. A global database of food and nutrient consumption. Bull. World Health Organ. 2016;94:931–934.

[29] Micha R, Coates J, Leclercq C, et al. Global Dietary Surveillance: Data Gaps and Challenges. Food Nutr. Bull. 2018;39:175–205.

[30] Caribbean Community Secretariat. Revised Common External Tariff of the Caribbean Community [Internet]. 2018 [cited 2019 Oct 8]. Available from: https://caricom.org/documents/16273-revised_cet_of_caricom_hs_2017_revised_11_april_2018_(for_link).pdf.

[31] Central Intelligence Agency. Central America: Barbados [Internet]. World Factb. [cited 2018 Nov 23]. Available from: https://www.cia.gov/librarY/publications/the-world-factbook/geos/bb.html.

[32] Howitt C, Hambleton IR, Rose AMC, et al. Social distribution of diabetes, hypertension and related risk factors in Barbados: a cross-sectional study. BMJ Open. 2015;5:e008869.

[33] Harris RM, Rose AMC, Hambleton IR, et al. Sodium and potassium excretion in an adult Caribbean population of African descent with a high burden of cardiovascular disease. BMC Public Health [Internet]. 2018 [cited 2018 Aug 31];18. Available from: https://bmcpublichealth.biomedcentral.com/articles/10.1186/s12889-018-5694-0.

[34] Raper N, Perloff B, Ingwersen L, et al. An overview of USDA's Dietary Intake Data System. J. Food Compos. Anal. 2004;17:545–555.

[35] Nutribase Pro. E. Muirwood Drive, Phoenix, Arizona, USA: Cybersoft Inc.; 2016.

[36] Lachat C, Hawwash D, Ocké MC, et al. Strengthening the Reporting of Observational Studies in Epidemiology—Nutritional Epidemiology (STROBE-nut): An Extension of the STROBE Statement. PLOS Med. 2016;13:e1002036.

[37] Chambers S, Barton KL, Albani V, et al. Identifying dietary differences between Scotland and England: a rapid review of the literature. Public Health Nutr. 2017;20:2459–2477.

[38] World Bank. World Bank Climate Change Knowledge Portal: Barbados [Internet]. [cited 2019 Oct 9]. Available from: https://climateknowledgeportal.worldbank.org/country/barbados.

[39] Jackson M, Walker S, Forrester T, et al. Social and dietary determinants of body mass index of adult Jamaicans of African origin. Eur. J. Clin. Nutr. 2003;57:621–627.

[40] Han E, Powell LM. Consumption patterns of sugar-sweetened beverages in the United States. J. Acad. Nutr. Diet. 2013;113:43–53.

[41] Newens KJ, Walton J. A review of sugar consumption from nationally representative dietary surveys across the world. J. Hum. Nutr. Diet. Off. J. Br. Diet. Assoc. 2016;29:225–240.

For peer review only - http://bmjopen.bmj.com/site/about/guidelines.xhtml

[42] Louie JCY, Moshtaghian H, Rangan AM, et al. Intake and sources of added sugars among Australian children and adolescents. Eur. J. Nutr. 2016;55:2347–2355.

[43] Caro JC, Corvalán C, Reyes M, et al. Chile's 2014 sugar-sweetened beverage tax and changes in prices and purchases of sugar-sweetened beverages: An observational study in an urban environment. PLOS Med. 2018;15:e1002597.

[44] Government of Barbados. Excise Tax (Amendment) (No.) Regulations, 2017 [Internet]. Nov 29, 2017. Available from: https://www.barbadosparliament.com/uploads/sittings/attachments/52aa3985de8d4ef6f99b91d346914ba5.pdf.

[45] Chaloupka F, Powell L. Using Fiscal Policy to Promote Health: Taxing Tobacco, Alcohol, and Sugary Beverages. Backgr. Pap. Task Force Fisc. Policy Health. 2018;25.

[46] Public Health England. The Eatwell Guide [Internet]. 2018. p. 12. Available from: https://www.gov.uk/government/publications/the-eatwell-guide.

[47] Ministry of Health. Food Based Dietary Guidelines for Barbados [Internet]. 2017 [cited 2019 Jan 11]. Available from: http://www.fao.org/3/I9680EN/i9680en.pdf.

[48] Government of Saint Christopher and Nevis. Excise Tax Act. Off. Gaz. 2010;52.

[49] Government of Bolivia L. Actualización de las alícuotas específicas del impuesto a los consumos específicos (ICE) para la gestión. 2018;5.

[50] South African Revenue Service (SARS). Schedules to the Customs and Excise Act, 1964 (Tariff Book) [Internet]. Sched. No 1 Part 7A. Available from: https://www.sars.gov.za/AllDocs/LegalDoclib/SCEA1964/LAPD-LPrim-Tariff-2012-13b%20-%20Schedule%20No%201%20Part%207A.pdf.

For peer review only

For peer review only - http://bmjopen.bmj.com/site/about/guidelines.xhtml

For peer review only

For peer review only - http://bmjopen.bmj.com/site/about/guidelines.xhtml

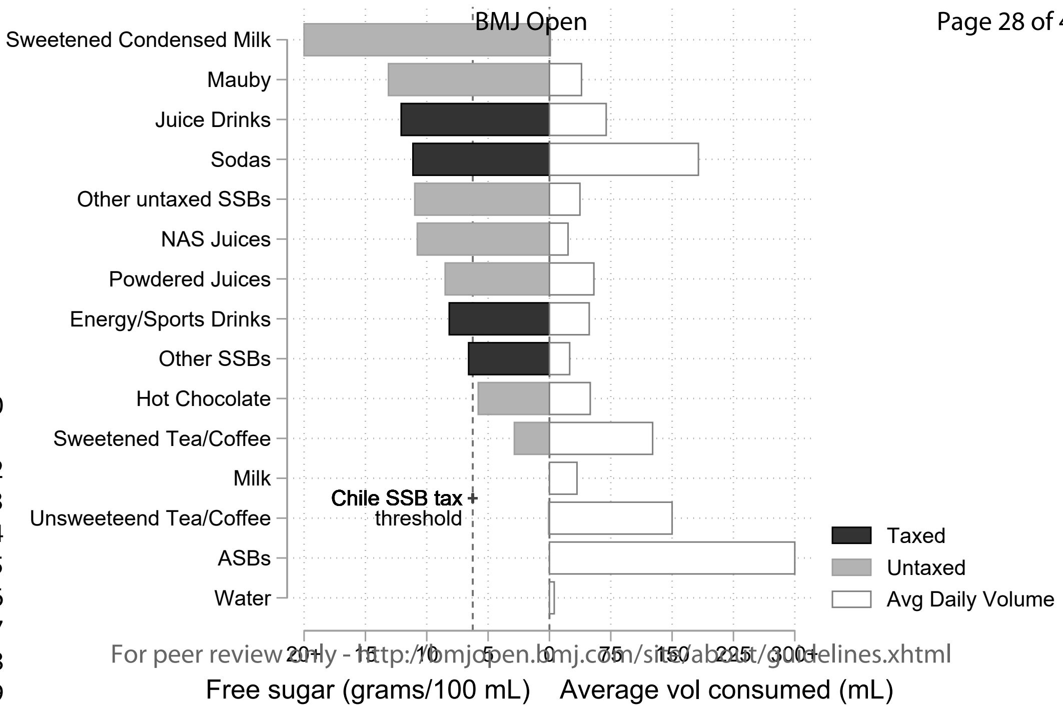

For peer review only - http://bmjopen.bmj.com/site/about/guidelines.xhtml

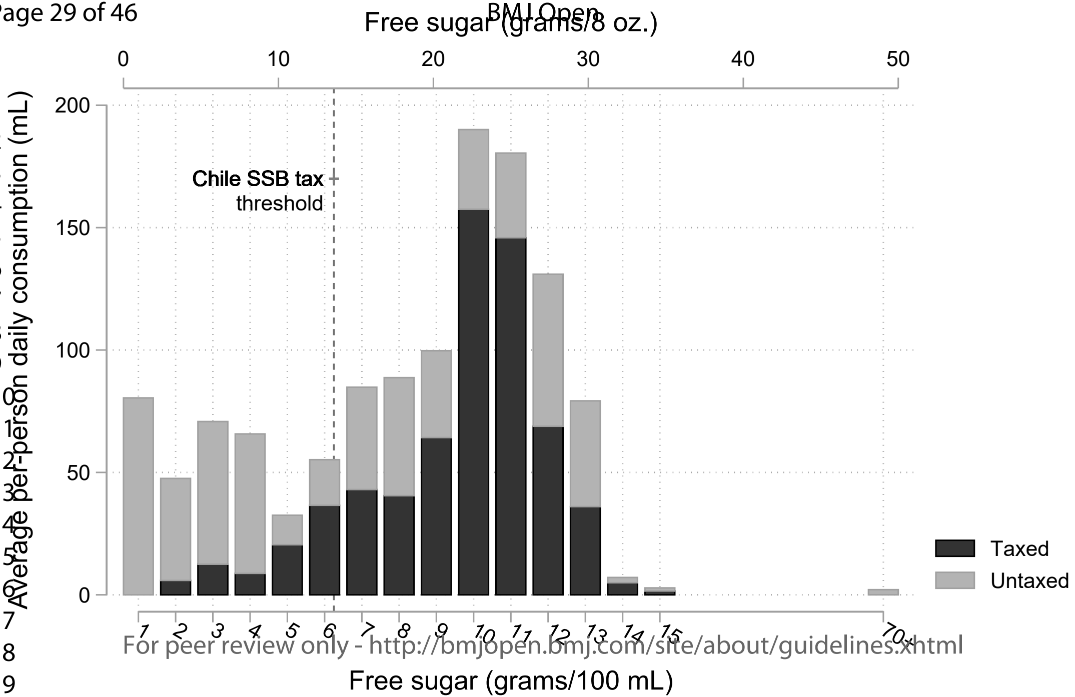

For peer review only - http://bmjopen.bmj.com/site/about/guidelines.xhtml

Appendix Files:

1. **Appendix Table 1:** Beverage category definitions
2. **Appendix Table 2:** STROBE-nut: An extension of the STROBE statement for nutritional epidemiology
3. **Appendix Table 3:** Products by taxable status and free sugar concentration levels (with specific brands as exemplars)
4. **Appendix Text 1:** Definition of beverage categories and nutrient composition
5. **Appendix Text 2:** Sugar concentration by product types
6. **Appendix Figure 1:** Distribution of SSB consumption in mean daily servings/capita, given any SSB consumption, Barbados 2012-2013: the Barbados Salt Intake Study
7. **Appendix Figure 2**: Per-person mean Total Energy Intake (TEI) attributable to SSB-related free sugars consumption (%), given any consumption, Barbados 2012-2013: the Barbados Salt Intake Study
8. **Appendix Figure 3:** Mean per-person free sugar consumed from soft drinks amongst adults aged 25-64, by product type and taxable status, Barbados 2012-2013: the Barbados Salt Intake Study

For peer review only

Appendix Table 1: Beverage category definitions

Main Categories	Taxable status	Level I Category	Level II Categories	Examples of frequently consumed drinks
SSBs	Taxed SSBs	Soda	Soda	Coca Cola, Frutee, Fanta, Sprite, Pepsi
		Juice Drinks	Juice Drinks	Pinehill Dairy Juice drinks, Fruta
		Energy/Sports/Malt Drinks	Malt Drinks	VitaMalt
			Energy Drinks	Redbull, Monster
			Sports Drinks	Gatroade, Lucozade
		Other taxed SSBs	Flavored SSB water	Cranwater
			Flavoured Dairy	Indulgence Milk
			Other taxed SSBs	Store-bought iced tea, Ensure, Supligen, seamoss
	Untaxed SSBs	Mauby*	Mauby*	Mauby*
		Powdered Juice Drinks	Powdered Juice Drinks	Tang, turbo, koolaid
		Hot Chocolate	Powdered hot chocolate	Milo, Nestle
		Sweetened tea/coffee	Homemade sweetened tea/coffee	coffee, tea or iced tea w/ added sugar or sweetened condensed milk
		Sweetened condensed milk	Sweetened condensed milk	Sweetened condensed milk consumed with cereal or cream of wheat as a milk substitute
		Other untaxed SSBs	Homemade SSBs juice drinks	sugar-sweetened homemade smoothies
				sugar-sweetened homemade juices
			Other untaxed SSBs	snowcone, milkshake, purchased sweetened coffee drinks (lattes, mochas etc)
Non-SSBs	Untaxed non-SSBs	Water	Water	Tap water, bottled water, soda water
		NAS juice (no added sugar)	NAS Juice	NAS Pinehill Dairy, Dewlands, Ceres
			Homemade non-SSBs	no-sugar added homemade juices
				no-sugar added homemade smoothies
		Other non-SSB	no added sugar coffee/tea	Coffee/tea with no added sugar
		Milk	Milk	Milk
		ASB	Diet Soda	Diet Coke

9. *Although mauby could be considered a homemade drink it can also be purchased as a syrup or a ready-made drink, and as such we report it as a separate category under SSBs.

For peer review only

For peer review only - http://bmjopen.bmj.com/site/about/guidelines.xhtml

Appendix Table 2: STROBE-nut: An extension of the STROBE statement for nutritional epidemiology (Lachat C et al., 2016)

Item	Item nr	STROBE recommendations	Extension for Nutritional Epidemiology studies (STROBE-nut)	Reported on page #
**Title and abstract**	1	(a) Indicate the study's design with a commonly used term in the title or the abstract.<br><br>(b) Provide in the abstract an informative and balanced summary of what was done and what was found.	**nut-1**State the dietary/nutritional assessment method(s) used in the title, abstract, or keywords.	**Abstract**
**Introduction**				
Background rationale	2	Explain the scientific background and rationale for the investigation being reported.		1-2
Objectives	3	State specific objectives, including any pre-specified hypotheses.		3
**Methods**				
Study design	4	Present key elements of study design early in the paper.		3
Settings	5	Describe the setting, locations, and relevant dates, including periods of recruitment, exposure, follow-up, and data collection.	**nut-5**Describe any characteristics of the study settings that might affect the dietary intake or nutritional status of the participants, if applicable.	3
Participants	6	a) Cohort study—Give the eligibility criteria, and the sources and methods of selection of participants. Describe methods of follow-up.<br><br>Case-control study—Give the eligibility criteria, and the sources and methods of case ascertainment and control selection. Give the rationale for the choice of cases and controls.<br><br>Cross-sectional study—Give the eligibility criteria, and the sources and methods of selection of participants.<br><br>(b) Cohort study—For matched studies, give matching criteria and number of exposed and unexposed.	**nut-6** Report particular dietary, physiological or nutritional characteristics that were considered when selecting the target population.	3-4

For peer review only - http://bmjopen.bmj.com/site/about/guidelines.xhtml

Item	Item nr	STROBE recommendations	Extension for Nutritional Epidemiology studies (STROBE-nut)	Reported on page #
		Case-control study—For matched studies, give matching criteria and the number of controls per case.		
Variables	7	Clearly define all outcomes, exposures, predictors, potential confounders, and effect modifiers. Give diagnostic criteria, if applicable.	**nut-7.1**Clearly define foods, food groups, nutrients, or other food components.	**4-5**
			**nut-7.2** When using dietary patterns or indices, describe the methods to obtain them and their nutritional properties.	**3**
Data sources - measurements	8	For each variable of interest, give sources of data and details of methods of assessment (measurement).Describe comparability of assessment methods if there is more than one group.	**nut-8.1** Describe the dietary assessment method(s), e.g., portion size estimation, number of days and items recorded, how it was developed and administered, and how quality was assured. Report if and how supplement intake was assessed.	**3-4**
			**nut-8.2** Describe and justify food composition data used. Explain the procedure to match food composition with consumption data. Describe the use of conversion factors, if applicable.	**3-4**
			**nut-8.3** Describe the nutrient requirements, recommendations, or dietary guidelines and the evaluation approach usedto compare intake with the dietary reference values, if applicable.	**5**
			**nut-8.4** When using nutritional biomarkers, additionally use the STROBE Extension for Molecular Epidemiology (STROBE-ME). Report the type of biomarkers used and their usefulness as dietary exposure markers.	**NA**
			**nut-8.5** Describe the assessment of nondietary data (e.g., nutritional status and influencing factors) and timing of the assessment of these variables in relation to dietary assessment.	**4-5**

For peer review only

**Item**	**Item nr**	**STROBE recommendations**	**Extension for Nutritional Epidemiology studies (STROBE-nut)**	**Reported on page #**
			**nut-8.6** Report on the validity of the dietary or nutritional assessment methods and any internal or external validation used in the study, if applicable.	**3-4**
Bias	9	Describe any efforts to address potential sources of bias.	**nut-9** Report how bias in dietary or nutritional assessment was addressed, e.g., misreporting, changes in habits as a result of being measured, or data imputation from other sources	**3-4 and Appendix Text 1-2**
Study Size	10	Explain how the study size was arrived at.		**3-4**
Quantitative variables	11	Explain how quantitative variables were handled in the analyses. If applicable, describe which groupings were chosen and why.	**nut-11** Explain categorization of dietary/nutritional data (e.g., use of N-tiles and handling of nonconsumers) and the choice of reference category, if applicable.	**4**
Statistical Methods	12	(a) Describe all statistical methods, including those used to control for confounding	**nut-12.1** Describe any statistical method used to combine dietary or nutritional data, if applicable.	**3, 4**
		(b) Describe any methods used to examine subgroups and interactions.	**nut-12.2** Describe and justify the method for energy adjustments, intake modeling, and use of weighting factors, if applicable.	**3,4, 6**
		(c) Explain how missing data were addressed.	**nut-12.3** Report any adjustments for measurement error, i.e,. from a validity or calibration study.	
		(d) Cohort study—If applicable, explain how loss to follow-up was addressed.		
		Case-control study—If applicable, explain how matching of cases and controls was addressed.		
		Cross-sectional study—If applicable, describe analytical methods taking account of sampling strategy.		
		(e) Describe any sensitivity analyses.		
**Results**				
Participants	13	(a) Report the numbers of individuals at each stage of the study—e.g., numbers potentially eligible, examined for eligibility, confirmed eligible, included	**nut-13** Report the number of individuals excluded based on missing, incomplete or implausible dietary/nutritional data.	**4**

For peer review only - http://bmjopen.bmj.com/site/about/guidelines.xhtml

Item	Item nr	STROBE recommendations	Extension for Nutritional Epidemiology studies (STROBE-nut)	Reported on page #
		in the study, completing follow-up, and analyzed.<br><br>(b) Give reasons for non-participation at each stage.<br><br>(c) Consider use of a flow diagram.		
Descriptive data	14	(a) Give characteristics of study participants (e.g., demographic, clinical, social) and information on exposures and potential confounders<br><br>(b) Indicate the number of participants with missing data for each variable of interest<br><br>(c) Cohort study—Summarize follow-up time (e.g., average and total amount)	**nut-14** Give the distribution of participant characteristics across the exposure variables if applicable. Specify if food consumption of total population or consumers only were used to obtain results.	**Table 1, p5**
Outcome data	15	Cohort study—Report numbers of outcome events or summary measures over time.<br><br>Case-control study—Report numbers in each exposure category, or summary measures of exposure.<br><br>Cross-sectional study—Report numbers of outcome events or summary measures.		
Main results	16	(a) Give unadjusted estimates and, if applicable, confounder-adjusted estimates and their precision (e.g., 95% confidence interval).<br><br>Make clear which confounders were adjusted for and why they were included.<br><br>(b) Report category boundaries when continuous variables were categorized.<br><br>(c) If relevant, consider translating estimates of relative risk into absolute risk for a meaningful time period.	**nut-16** Specify if nutrient intakes are reported with or without inclusion of dietary supplement intake, if applicable.	**NA**
Other analyses	17	Report other analyses done—e.g., analyses of subgroups and interactions and sensitivity analyses.	**nut-17**Report any sensitivity analysis (e.g., exclusion of misreporters or	**NA**

For peer review only - http://bmjopen.bmj.com/site/about/guidelines.xhtml

**Item**	**Item nr**	**STROBE recommendations**	**Extension for Nutritional Epidemiology studies (STROBE-nut)**	**Reported on page #**
			outliers) and data imputation, if applicable.	
**Discussion**				
Key results	18	Summarize key results with reference to study objectives.		7
Limitation	19	Discuss limitations of the study, taking into account sources of potential bias or imprecision. Discuss both direction and magnitude of any potential bias.	**nut-19** Describe the main limitations of the data sources and assessment methods used and implications for the interpretation of the findings.	**8**
Interpretation	20	Give a cautious overall interpretation of results considering objectives, limitations, multiplicity of analyses, results from similar studies, and other relevant evidence.	**nut-20** Report the nutritional relevance of the findings, given the complexity of diet or nutrition as an exposure.	**8-10**
Generalizability	21	Discuss the generalizability (external validity) of the study results.		10
**Other information**				
Funding	22	Give the source of funding and the role of the funders for the present study and, if applicable, for the original study on which the present article is based.		**16**
Ethics			**nut-22.1**Describe the procedure for consent and study approval from ethics committee(s).	**16**
Supplementary material			**nut-22.2** Provide data collection tools and data as online material or explain how they can be accessed.	**Appendix File**

For peer review only - http://bmjopen.bmj.com/site/about/guidelines.xhtml

Appendix Table 3: Products by taxable status and free sugar concentration levels (with specific brands as exemplars)

Free sugar concentration	Taxable status	Products
**1-2.9**	Taxed	NA
	Untaxed	SSB coffee & tea, soymilk
**3-4.9**	Taxed	Flavoured water (Cranwater)
	Untaxed	Hot chocolate, soy milk, SSB coffee & tea, powdered juice (Mak-C)
**5-6.9**	Taxed	Sports drinks (Powerade), soda (Frutee - ginger ale flavor), flavored milk
	Untaxed	Powdered juice (Tang), SSB coffee & tea, powdered milk
**7-.8.9**	Taxed	Malt, Energy drinks (Plus), juice drinks
	Untaxed	Powdered juice (Turbo), SSB coffee & tea, iced tea
**9-10.9**	Taxed	Soda (Sprite, Busta), flavored milk
	Untaxed	Powdered juice (Koolaid), homemade sweet juice, NAS juice
**11.-12.9**	Taxed	Soda (Coca Cola, Frutee), juice drinks (Pinehill Dairy, Fruta)
	Untaxed	Lemonade, NAS juice, homemade juices/shakes/punch
**13-14.9**	Taxed	Juice drinks (Pinehill Dairy, Fruta), soda (Frutee, Ju-C)
	Untaxed	Mauby, homemade sweetened juice, NAS juice (Pinehill Dairy), SSB coffee & tea
**15-16.9**	Taxed	Soda, juice drinks
	Untaxed	SSB coffee & tea
**70+**	Taxed	NA
	Untaxed	Sweetened condensed milk

For peer review only - http://bmjopen.bmj.com/site/about/guidelines.xhtml

Appendix Text 1: Definition of beverage categories and nutrient composition

We categorized drinks according to the categories summarized in Appendix Table 2.

Nutribase includes nutrient information from the United States Department of Agriculture (USDA) and Canadian food composition databases. For products not included in Nutribase (e.g. local and regional brands of sodas, juices; internationally produced beverages imported from South Africa, Turkey, etc) we assigned nutrient information (sugars in grams and total calories in kilocalories) based on nutrient label data collected from product packages in stores and from websites. When a specific brand and flavour were reported in the dietary recall, we used nutrient information from the corresponding product. When no flavour was reported, we used the mean nutrient values across a range of available flavours. We relied on Nutribase nutrient information for available international brands (e.g. Coke, Sprite, etc). When no brand was reported in the dietary recall, we used the mean nutrient information for that beverage category.

For powdered drinks (powdered juices and hot chocolate) we used packet instructions to estimate reconstituted levels. Most powdered drinks reported in the recalls already include sugar and do not require additional sugar to be added. While people may add additional sugar, this was not included as a prompt in the standard 24-hr dietary recall, so our estimates of sugar intake from powdered drinks may be an underestimate.

For powdered milk we assumed a 1:5 dilution ratio and corrected levels of total calories and sugars accordingly (since the product was previously entered as undiluted powdered milk in Nutribase).

For homemade SSBs, a previous Barbados-based study used the weighed recipe approach to estimate nutrient content for three popular drinks: mauby, ginger beer and lemonade [51]. For other homemade drinks, we used the recipes that participants reported to identify similar products within Nutribase. Participants had been prompted for recipes and we used the ingredients to identify similar products within Nutribase. For homemade SSBs (smoothies and juice drinks), we categorized these as "fruit punch drink "pina colada," "blended smoothie, banana, oats, milk, honey, yogurt," "flavored milks," "pina colada," "blended shake, milkshake vanilla," "mixed berry fruit smoothie," "fruit 'n' yogurt smoothie, strawberry kiwi," "tropical fruit smoothie," "golden apple juice," "lemonade," "juice apple & cherry juice," "island guava drink," "orange flavor drink" "passion fruit juice," "dock, boiled (sorrel)," "mixed fruit juice," or "grape juice" as appropriate.

For homemade non-SSB (no added sugar smoothies and juices), we categorized these as "blended carrot, beet, celery, cucumber, apple juice without sugar," "cranberry juice," "carrot juice," "V8 60% vegetable juice, V-Lite," "aloe vera juice, " "mango juice," "orange juice, unsweetened," "lemon juice, raw," "passion fruit juice, raw," "soy milk," or "mandarin papaya drink" as appropriate.

Mauby is a local bark that is boiled with water and sugar to make a sweet drink (and can also be bought as a ready-made syrup and diluted at home or purchased ready-to-drink).

Sorrel is a flower (similar to hibiscus) that is used to make a sweetened drink. Golden apples are a fruit that are used to make a juice (often sweetened).

We excluded snowcones, as we considered these to be a dessert and not a drink.

Several drinks were categorized within Nutribase as "Pina coladas" although upon review these were identified to be homemade punches or smoothies. The sugar and total calories content of these four observation were rescaled, with pineapple punch and coconut punch re-scaled based on "fruit punch

For peer review only - http://bmjopen.bmj.com/site/about/guidelines.xhtml

drink" and "smoothie homemade" and "mango shake, homemade blended almond milk" re-scaled based on ("blended smoothie - banana oats milk honey yogurt").

To exclude galactose and lactose sugars, we subtracted these from total sugars. Where Nutribase did not automatically assign lactose/galactose sugar content to milk products, we assumed all sugars were from lactose/galactose in no added sugar milk products.

When sweetened condensed milk was reported with coffee/tea, we estimated the total sugar concentration per quantity of coffee/tea consumed and reported this under "sweetened tea/coffee" rather than "sweetened condensed milk." When consumption was reported with cereal or cream of wheat in place of regular milk we reported this under "sweetened condensed milk."

Throughout this report, "SSBs" refer to both taxed and untaxed SSBs (excluding non-SSBs), while "soft drinks" refer to both SSBs and non-SSBs. Some non-SSBs (such as no added sugar juice) contain free sugars. To clarify when non-SSBs are included, we refer to "soft-drinks" rather than "SSBs."

For participants with more than two recalls (n=1) we used only the first two recalls, assuming that reporting quality may have changed with repeated exposure to the survey instrument.

We converted all reported beverage volumes into milliliters.

Appendix Text 2: Sugar concentration by product types

The sugar concentration of some product types varied greatly, such as for home-prepared SSB tea/coffee with reported consumption at almost every sugar concentration level. Other product types were more narrowly defined (such as flavoured water, which was only found in the 3-4.9 gr/100mL category). Most of the sweetest products (13+ gr/100mL) were locally or regionally produced fruit drinks or sodas. Some flavours of no added sugar juice (non-SSBs) had a higher sugar concentration than juice drinks (SSBs), and some flavours of sodas had notably lower levels of sugar concentration than other flavours under the same brand. Sweetened condensed milk consumed as a milk substitute with cereal or cream of wheat was the only product with a sugar concentration greater than 17+ gr/100mL.

For peer review only - http://bmjopen.bmj.com/site/about/guidelines.xhtml

Appendix Figure 1: Distribution of SSB consumption in mean daily servings/capita, given any SSB consumption, Barbados 2012-2013: the Barbados Salt Intake Study

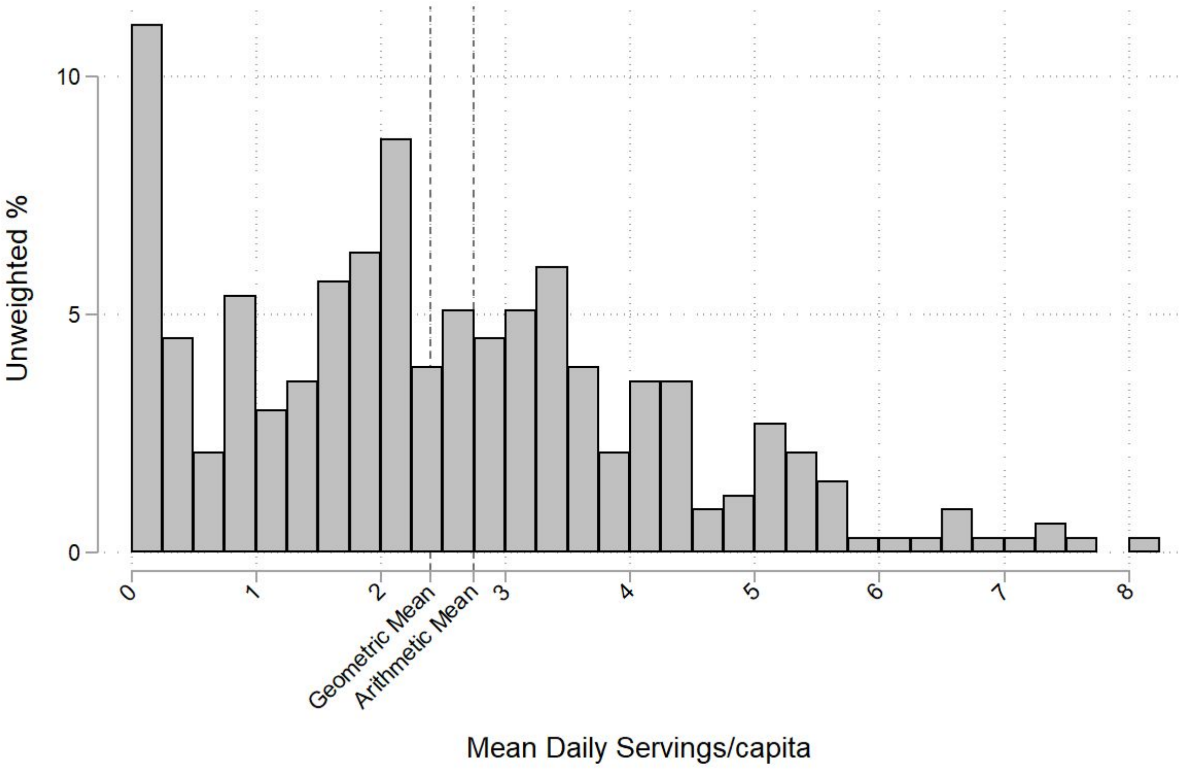

Appendix Figure 2: Per-person mean Total Energy Intake (TEI) attributable to SSB-related free sugars consumption (%), given any consumption, Barbados 2012-2013: the Barbados Salt Intake Study

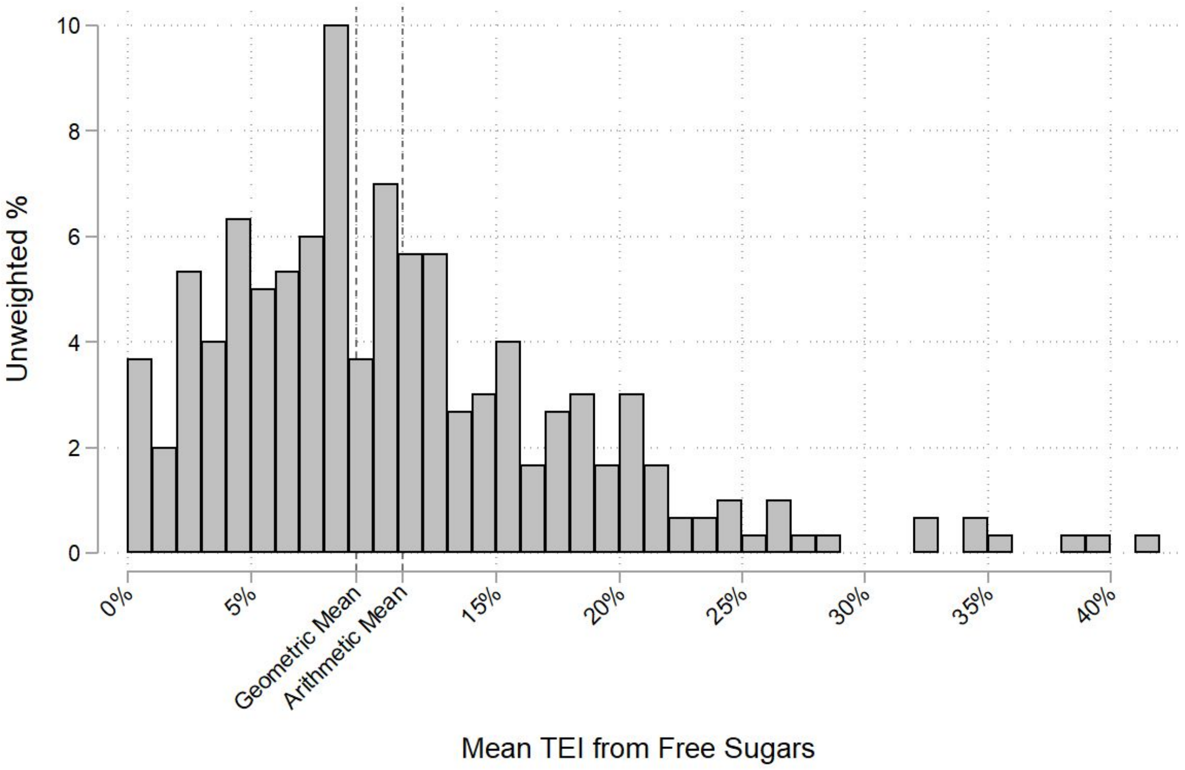

For peer review only - http://bmjopen.bmj.com/site/about/guidelines.xhtml

Appendix Figure 3: Mean per-person free sugar consumed from soft drinks amongst adults aged 25-64, by product type and taxable status, Barbados 2012-2013: the Barbados Salt Intake Study

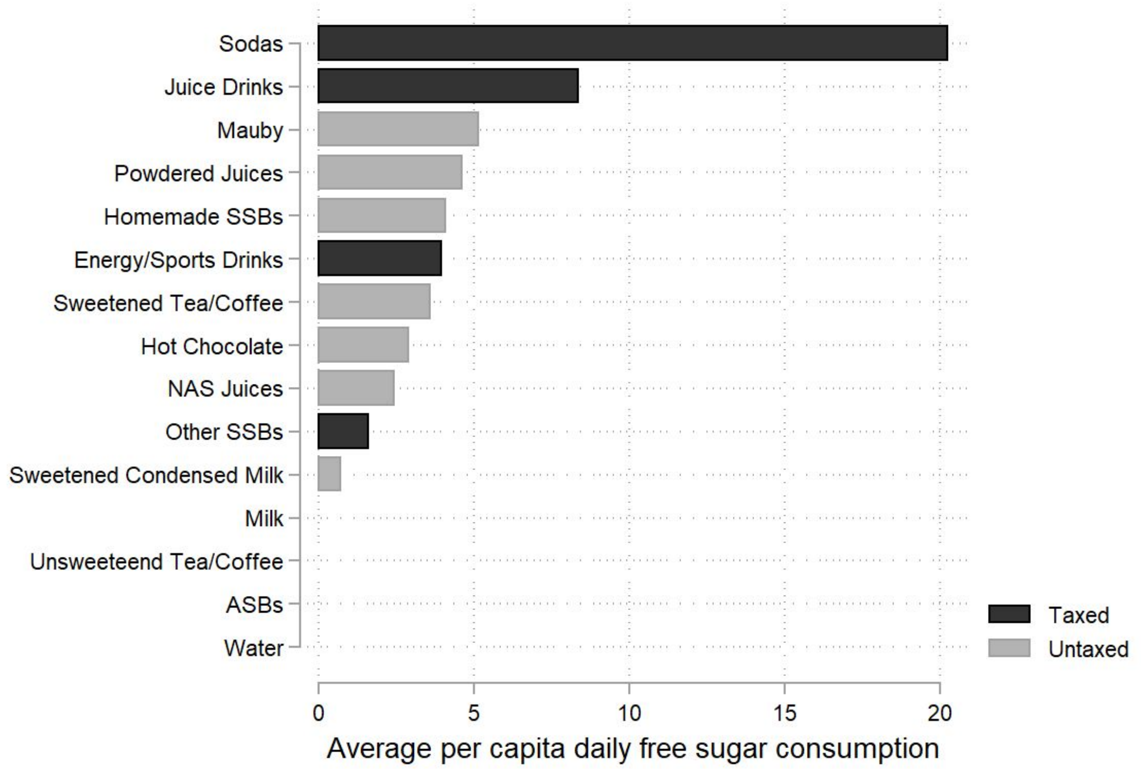

For peer review only - http://bmjopen.bmj.com/site/about/guidelines.xhtml

Research Checklist: STROBE-nut: An extension of the STROBE statement for nutritional epidemiology (Lachat C et al., 2016)

Item	Item nr	STROBE recommendations	Extension for Nutritional Epidemiology studies (STROBE-nut)	Reported on page #
**Title and abstract**	1	(a) Indicate the study's design with a commonly used term in the title or the abstract.<br><br>(b) Provide in the abstract an informative and balanced summary of what was done and what was found.	**nut-1**State the dietary/nutritional assessment method(s) used in the title, abstract, or keywords.	**Abstract**
**Introduction**				
Background rationale	2	Explain the scientific background and rationale for the investigation being reported.		1-2
Objectives	3	State specific objectives, including any pre-specified hypotheses.		3
**Methods**				
Study design	4	Present key elements of study design early in the paper.		3
Settings	5	Describe the setting, locations, and relevant dates, including periods of recruitment, exposure, follow-up, and data collection.	**nut-5**Describe any characteristics of the study settings that might affect the dietary intake or nutritional status of the participants, if applicable.	3
Participants	6	a) Cohort study—Give the eligibility criteria, and the sources and methods of selection of participants. Describe methods of follow-up.<br><br>Case-control study—Give the eligibility criteria, and the sources and methods of case ascertainment and control selection. Give the rationale for the choice of cases and controls.<br><br>Cross-sectional study—Give the eligibility criteria, and the sources and methods of selection of participants.<br><br>(b) Cohort study—For matched studies, give matching criteria and number of exposed and unexposed.	**nut-6** Report particular dietary, physiological or nutritional characteristics that were considered when selecting the target population.	3-4

For peer review only

Item	Item nr	STROBE recommendations	Extension for Nutritional Epidemiology studies (STROBE-nut)	Reported on page #
		Case-control study—For matched studies, give matching criteria and the number of controls per case.		
Variables	7	Clearly define all outcomes, exposures, predictors, potential confounders, and effect modifiers. Give diagnostic criteria, if applicable.	**nut-7.1**Clearly define foods, food groups, nutrients, or other food components.	**4-5**
			**nut-7.2** When using dietary patterns or indices, describe the methods to obtain them and their nutritional properties.	**3**
Data sources - measurements	8	For each variable of interest, give sources of data and details of methods of assessment (measurement).Describe comparability of assessment methods if there is more than one group.	**nut-8.1** Describe the dietary assessment method(s), e.g., portion size estimation, number of days and items recorded, how it was developed and administered, and how quality was assured. Report if and how supplement intake was assessed.	**3-4**
			**nut-8.2** Describe and justify food composition data used. Explain the procedure to match food composition with consumption data. Describe the use of conversion factors, if applicable.	**3-4**
			**nut-8.3** Describe the nutrient requirements, recommendations, or dietary guidelines and the evaluation approach usedto compare intake with the dietary reference values, if applicable.	**5**
			**nut-8.4** When using nutritional biomarkers, additionally use the STROBE Extension for Molecular Epidemiology (STROBE-ME). Report the type of biomarkers used and their usefulness as dietary exposure markers.	**NA**
			**nut-8.5** Describe the assessment of nondietary data (e.g., nutritional status and influencing factors) and timing of the assessment of these variables in relation to dietary assessment.	**4-5**

For peer review only

Item	Item nr	STROBE recommendations	Extension for Nutritional Epidemiology studies (STROBE-nut)	Reported on page #
			**nut-8.6** Report on the validity of the dietary or nutritional assessment methods and any internal or external validation used in the study, if applicable.	**3-4**
Bias	9	Describe any efforts to address potential sources of bias.	**nut-9** Report how bias in dietary or nutritional assessment was addressed, e.g., misreporting, changes in habits as a result of being measured, or data imputation from other sources	**3-4 and Appendix Text 1-2**
Study Size	10	Explain how the study size was arrived at.		**3-4**
Quantitative variables	11	Explain how quantitative variables were handled in the analyses. If applicable, describe which groupings were chosen and why.	**nut-11** Explain categorization of dietary/nutritional data (e.g., use of N-tiles and handling of nonconsumers) and the choice of reference category, if applicable.	**4**
Statistical Methods	12	(a) Describe all statistical methods, including those used to control for confounding<br>(b) Describe any methods used to examine subgroups and interactions.<br>(c) Explain how missing data were addressed.<br>(d) Cohort study—If applicable, explain how loss to follow-up was addressed.<br>Case-control study—If applicable, explain how matching of cases and controls was addressed.<br>Cross-sectional study—If applicable, describe analytical methods taking account of sampling strategy.<br>(e) Describe any sensitivity analyses.	**nut-12.1** Describe any statistical method used to combine dietary or nutritional data, if applicable.<br>**nut-12.2** Describe and justify the method for energy adjustments, intake modeling, and use of weighting factors, if applicable.<br>**nut-12.3** Report any adjustments for measurement error, i.e,. from a validity or calibration study.	**3, 4**<br>**3,4, 6**
**Results**				
Participants	13	(a) Report the numbers of individuals at each stage of the study—e.g., numbers potentially eligible, examined for eligibility, confirmed eligible, included	**nut-13** Report the number of individuals excluded based on missing, incomplete or implausible dietary/nutritional data.	**4**

For peer review only

Item	Item nr	STROBE recommendations	Extension for Nutritional Epidemiology studies (STROBE-nut)	Reported on page #
		in the study, completing follow-up, and analyzed.<br><br>(b) Give reasons for non-participation at each stage.<br><br>(c) Consider use of a flow diagram.		
Descriptive data	14	(a) Give characteristics of study participants (e.g., demographic, clinical, social) and information on exposures and potential confounders<br><br>(b) Indicate the number of participants with missing data for each variable of interest<br><br>(c) Cohort study—Summarize follow-up time (e.g., average and total amount)	**nut-14** Give the distribution of participant characteristics across the exposure variables if applicable. Specify if food consumption of total population or consumers only were used to obtain results.	**Table 1, p5**
Outcome data	15	Cohort study—Report numbers of outcome events or summary measures over time.<br><br>Case-control study—Report numbers in each exposure category, or summary measures of exposure.<br><br>Cross-sectional study—Report numbers of outcome events or summary measures.		
Main results	16	(a) Give unadjusted estimates and, if applicable, confounder-adjusted estimates and their precision (e.g., 95% confidence interval).<br><br>Make clear which confounders were adjusted for and why they were included.<br><br>(b) Report category boundaries when continuous variables were categorized.<br><br>(c) If relevant, consider translating estimates of relative risk into absolute risk for a meaningful time period.	**nut-16** Specify if nutrient intakes are reported with or without inclusion of dietary supplement intake, if applicable.	**NA**
Other analyses	17	Report other analyses done—e.g., analyses of subgroups and interactions and sensitivity analyses.	**nut-17**Report any sensitivity analysis (e.g., exclusion of misreporters or	**NA**

For peer review only

Item	Item nr	STROBE recommendations	Extension for Nutritional Epidemiology studies (STROBE-nut)	Reported on page #
			outliers) and data imputation, if applicable.	
**Discussion**				
Key results	18	Summarize key results with reference to study objectives.		7
Limitation	19	Discuss limitations of the study, taking into account sources of potential bias or imprecision. Discuss both direction and magnitude of any potential bias.	**nut-19** Describe the main limitations of the data sources and assessment methods used and implications for the interpretation of the findings.	**8**
Interpretation	20	Give a cautious overall interpretation of results considering objectives, limitations, multiplicity of analyses, results from similar studies, and other relevant evidence.	**nut-20** Report the nutritional relevance of the findings, given the complexity of diet or nutrition as an exposure.	**8-10**
Generalizability	21	Discuss the generalizability (external validity) of the study results.		10
**Other information**				
Funding	22	Give the source of funding and the role of the funders for the present study and, if applicable, for the original study on which the present article is based.		**16**
Ethics			**nut-22.1**Describe the procedure for consent and study approval from ethics committee(s).	**16**
Supplementary material			**nut-22.2** Provide data collection tools and data as online material or explain how they can be accessed.	**Appendix File**

For peer review only

BMJ Open

Using nutritional survey data to inform the design of sugar-sweetened beverage taxes in low-resource contexts: a cross-sectional analysis based on data from an adult Caribbean population

Journal:	*BMJ Open*
Manuscript ID	bmjopen-2019-035981.R1
Article Type:	Original research
Date Submitted by the Author:	17-Apr-2020

Complete List of Authors:	Alvarado, Miriam; University of Cambridge, MRC Epidemiology Unit<br>Harris, Rachel; University of the West Indies at Cave Hill, Faculty of Medical Sciences<br>Rose, Angela; University of the West Indies at Cave Hill, George Alleyne Chronic Disease Research Centre, Caribbean Institute for Health Research; Epiconcept, Epidemiology Department<br>Unwin, Nigel; University of Cambridge, Global Diet and Activity Research, MRC Epidemiology Unit; University of Exeter Medical School, College of Medicine and Health<br>Hambleton, Ian; University of the West Indies at Cave Hill, George Alleyne Chronic Disease Research Centre, Caribbean Institute for Health Research<br>Imamura, Fumiaki; University of Cambridge, MRC Epidemiology Unit<br>Adams, J; University of Cambridge, Centre for Diet and Activity Research, MRC Epidemiology Unit

**Primary Subject Heading**:	Public health
Secondary Subject Heading:	Epidemiology, Global health, Health policy, Nutrition and metabolism
Keywords:	Health policy < HEALTH SERVICES ADMINISTRATION & MANAGEMENT, Nutrition < TROPICAL MEDICINE, Epidemiology < TROPICAL MEDICINE, Public health < INFECTIOUS DISEASES

SCHOLARONE™
Manuscripts

I, the Submitting Author has the right to grant and does grant on behalf of all authors of the Work (as defined in the below author licence), an exclusive licence and/or a non-exclusive licence for contributions from authors who are: i) UK Crown employees; ii) where BMJ has agreed a CC-BY licence shall apply, and/or iii) in accordance with the terms applicable for US Federal Government officers or employees acting as part of their official duties; on a worldwide, perpetual, irrevocable, royalty-free basis to BMJ Publishing Group Ltd ("BMJ") its licensees and where the relevant Journal is co-owned by BMJ to the co-owners of the Journal, to publish the Work in this journal and any other BMJ products and to exploit all rights, as set out in our licence.

The Submitting Author accepts and understands that any supply made under these terms is made by BMJ to the Submitting Author unless you are acting as an employee on behalf of your employer or a postgraduate student of an affiliated institution which is paying any applicable article publishing charge ("APC") for Open Access articles. Where the Submitting Author wishes to make the Work available on an Open Access basis (and intends to pay the relevant APC), the terms of reuse of such Open Access shall be governed by a Creative Commons licence – details of these licences and which Creative Commons licence will apply to this Work are set out in our licence referred to above.

Other than as permitted in any relevant BMJ Author's Self Archiving Policies, I confirm this Work has not been accepted for publication elsewhere, is not being considered for publication elsewhere and does not duplicate material already published. I confirm all authors consent to publication of this Work and authorise the granting of this licence.

For peer review only

Title: Using nutritional survey data to inform the design of sugar-sweetened beverage taxes in low-resource contexts: a cross-sectional analysis based on data from an adult Caribbean population

Authors

Miriam Alvarado (corresponding author)

Centre for Diet and Activity Research, MRC Epidemiology Unit, University of Cambridge, Cambridge, United Kingdom

mra47@cam.ac.uk

http://orcid.org/0000-0003-2864-9410

+44 7944524573

Rachel Harris, Faculty of Medical Sciences, The University of the West Indies, Cave Hill, Barbados,

rachel.harris@mycavehill.uwi.edu

Orchid identifier:0000-0002-5205-4989

Angela Rose, George Alleyne Chronic Disease Research Centre, Caribbean Institute for Health Research, The University of the West Indies, Bridgetown, Barbados, angela.rose@epi-research.com;

Epidemiology Department, Epiconcept, Paris, France

https://orcid.org/0000-0002-2493-8082

Nigel Unwin, Global Diet and Activity Research, MRC Epidemiology Unit, University of Cambridge, Cambridge, United Kingdom, nigel.unwin@mrc-epid.cam.ac.uk;

University of Exeter Medical School, College of Medicine and Health, Truro, United Kingdom

https://orcid.org/0000-0002-1368-1648

For peer review only

For peer review only - http://bmjopen.bmj.com/site/about/guidelines.xhtml

Ian Hambleton, George Alleyne Chronic Disease Research Centre, Caribbean Institute for Health Research, The University of the West Indies, Bridgetown, Barbados, ian.hambleton@cavehill.uwi.edu; https://orcid.org/0000-0002-5638-9794

Fumiaki Imamura, MRC Epidemiology Unit, University of Cambridge, Cambridge, United Kingdom, Fumiaki.Imamura@mrc-epid.cam.ac.uk
https://orcid.org/0000-0002-6841-8396

Jean Adams, Centre for Diet and Activity Research, MRC Epidemiology Unit, University of Cambridge, Cambridge, United Kingdom, jma79@medschl.cam.ac.uk
http://orcid.org/0000-0002-5733-7830

Keywords: Health policy, Nutrition, Epidemiology, Prevention strategies, Public Health

Word count: 3246

Reference count: 50

Abstract word count: 252

For peer review only - http://bmjopen.bmj.com/site/about/guidelines.xhtml

Abstract

Objective

Sugar-sweetened beverage (SSB) taxes have been implemented widely. We aimed to use a pre-existing nutritional survey data to inform SSB tax design by assessing: 1) baseline consumption of SSBs and SSB-derived free sugars, 2) the percentage of SSB-derived free sugars that would be covered by a tax, and 3) the extent to which a tax would differentiate between high- and low-sugar SSBs. We evaluated these three considerations using pre-existing nutritional survey data in a developing economy setting.

Methods

We used data from a nationally representative cross-sectional survey in Barbados (2012-2013, prior to SSB tax implementation). Data were available on 334 adults (25-64 years) who completed two non-consecutive 24-hour dietary recalls. We estimated the prevalence of SSB consumption and its contribution to total energy intake, overall and stratified by taxable status. We assessed the percentage of SSB-derived free sugars subject to the tax and identified the consumption-weighted sugar concentration of SSBs, stratified by taxable status.

Findings

Accounting for sampling probability, 88.8% of adults (95%CI 85.1,92.5) reported SSB consumption, with a geometric mean of 2.4 servings/day (±2×standard deviation, 0.6,9.2) among SSB consumers. Sixty percent (95%CI 54.6, 65.4) of SSB-derived free sugars would be subject to the tax. The tax did not clearly differentiate between high- and low-sugar beverages.

Conclusion

Given high SSB consumption, targeting SSBs was a sensible strategy in this setting. A substantial percentage of free sugars from SSBs were not covered by the tax, reducing possible health benefits. The considerations proposed here may help policymakers to design more effective SSB taxes.

For peer review only - http://bmjopen.bmj.com/site/about/guidelines.xhtml

66 **Strengths and limitations of this study**

67 - A nationally representative dietary survey with two non-consecutive 24-hour dietary recalls allowed assessment of sugar-sweetened beverage (SSB) consumption patterns prior to the introduction of a tax on SSBs.

70 - Twenty-four-hour dietary recalls may be subject to reporting bias and may underestimate total SSB intake.

72 - Energy density (% of total energy intake) is reported to partially mitigate potential reporting biases.

74 - Data were not available on children, adolescents or adults over the age of 65.

75 - This is the first study that we are aware of to quantify the percentage of SSB-derived free sugars covered by a real-world SSB tax.

For peer review only

For peer review only - http://bmjopen.bmj.com/site/about/guidelines.xhtml

Background & Rationale

Background

The World Health Organization (WHO) has recommended limiting free sugar consumption to less than 10% of total energy intake (TEI).[1] Free sugars include sugars added to food and beverages, as well as sugars in fruit juices.[1] Sugar-sweetened beverages (SSBs) are a major source of free sugars, and consumption of SSBs is associated with higher risk of diabetes, certain cancers and obesity.[2–10]

Given these health risks, the WHO and others have recommended taxing SSBs to reduce consumption.[11–15] A number of countries (including many small island developing states (SIDS) and low- and middle-income countries) have introduced SSB taxes, at least in part for health reasons.[12,16–19]

However, these taxes vary widely in design.[16] In some settings, taxable products have been narrowly defined, whereas elsewhere they have been defined to include all soft drinks (even those containing no or small amounts of free sugars).[16,20] These differences are likely to have important health implications.[21]

The design (or amendment) of SSB taxes should be informed by local consumption patterns as much as possible. Commercial purchase data (such as Nielsen and Kantar consumer panels) have been used to assess SSB consumption patterns in the US and the UK, but these data are costly and unavailable in some settings.[22] In lower-resource settings in particular, it may be pragmatic to use pre-existing nutritional survey data to help inform context-specific policy design.[23,24] A recent review demonstrated that individual level dietary surveys have been conducted in at least 116 countries, representing 88.7% of the global 2010 adult population.[23,24] These nutritional survey data may provide a feasible way to inform the design or amendment of SSB taxes across a variety of settings. We highlight three ways in which these data may be used to improve the design of SSB taxation.

First, there is great heterogeneity in SSB consumption levels worldwide.[25] We suggest that SSB taxes are more likely to be effective at substantially reducing free sugar consumption (in absolute terms) in settings

For peer review only - http://bmjopen.bmj.com/site/about/guidelines.xhtml

in which SSB consumption levels are high and SSB-derived free sugars represent a high proportion of total energy intake.[26]

Second, we suggest that SSB taxes should cover a high proportion of regularly consumed SSBs, reducing substitution incentives.[12] If taxes are applied on a limited proportion of total SSBs consumed in a given population, the potential impact on health will be necessarily limited. If consumers substitute towards untaxed SSBs, health goals will be further undermined.

Finally, we suggest that SSB taxes should consistently differentiate between high- and low-sugar products.[27,28] If SSB taxes are not consistently applied on all high-sugar SSBs, health goals will be further undermined especially if consumers substitute towards high-sugar untaxed SSBs.

Box 1 summarizes these considerations.

Case Study: The Barbados SSB Tax

The Government of Barbados implemented a 10% SSB tax in 2015.[18] Taxable products (both imported and locally manufactured) were defined according to the Harmonized System (HS) tariff classifications and included soda, juice drinks, energy and sports drinks (tariff headings 20.09 and 22.02).[18,29] Some SSBs were not included in the tax definition, such as sugar-sweetened drink mixes (e.g. powdered juice and powdered hot chocolate) and sugar-sweetened syrups (e.g. mauby[1] syrups).[18]

A nationally representative nutritional survey was conducted in 2012-2013, well in advance of the introduction of the Barbados SSB tax in 2015. We revisited these data to assess the tax according to the considerations summarized in Box 1. We aimed to assess the following questions: 1) what were pre-tax SSB consumption levels (in terms of volume and contribution to TEI)? 2) what percentage of SSB-related

[1] Mauby is a local drink, which is typically sold either as a syrup to be reconstituted at home or as dried bark for home preparation

For peer review only - http://bmjopen.bmj.com/site/about/guidelines.xhtml

free sugars were covered by the tax? And 3) did the tax clearly differentiate between low and high-sugar beverages?

Methods

Study Design & Population

We used nutritional survey data from Barbados, a country with a population of 293,131 (2018 estimate) and $18,600 GDP/capita (2017 estimate).[30] Barbados is likely to share characteristics with low/middle-income and other SIDS settings (limited access to commercial sales data, a product-based definition of taxable products, etc.).

The data used in this study were from a sub-sample of the Health of the Nation study (HotN). The main HotN study was conducted between June 2012 and November 2013, with a response rate of 54% and final sample size of 1,234. Details of the overall sampling design, study recruitment and study procedures have been summarized elsewhere.[31,32] A sub-sample of 441 participants aged 25 to 64 were randomly selected from the HotN study to complete two non-consecutive in-person 24-hour dietary recalls.[33] In total, 368 participants (83%) consented to participate in the sub-study (for a combined response rate of 45%).

Each dietary recall was collected at home by a trained interviewer, using a standard multi-pass probing method, three-dimensional standardized food models and familiar measuring units.[34] Recalls were evenly distributed across quarters, with the exception of July-September when fewer recalls were conducted. The average time between the first and second recall was six days, and recalls were evenly distributed by day of the week. Data were processed using Nutribase Pro software.[35] Survey weights were used to reflect the clustered sampling design, to take into account the combined non-response rate and to match the age and sex distribution of the Barbados population as captured in the Barbados 2010 Census.[33]

We excluded participants with reported caloric intake less than 500 kcal/day or greater than 5000 kcal/day (n=5), those with missing covariate data (n=21), those with only one recall (n=1), and those with missing survey weights (n=7), leaving a total of 334 participants.

For peer review only - http://bmjopen.bmj.com/site/about/guidelines.xhtml

144 Ethics approval was given by the University of the West Indies Cavehill Institutional Review Board.

145 ***Patient and public involvement***

146 Participants were not involved in the design, conduct, reporting or dissemination of these analyses.

147 ***Measures of SSB Consumption***

148 We estimated the prevalence of SSB consumption, defined as those with any reported SSB consumption
149 on at least one day. Next, we estimated average volume consumed (mean SSB servings/day) amongst
150 SSB consumers (excluding those who did not report any SSB consumption). A serving was defined as
151 250 mL.[6] We reviewed each dietary recall and extracted product information for all reported SSBs.

152 Soft drinks were categorized based on whether they contained added sugars and whether they were
153 subject to the Barbados SSB tax. Taxed SSBs included soda, juice drinks, energy/sports/malt drinks and
154 other taxed SSBs; untaxed SSBs included sugar-sweetened powders (powdered juice drinks, hot
155 chocolate), sugar-sweetened syrups (mauby), sweetened tea/coffee, sweetened condensed milk and other
156 untaxed SSBs; and untaxed non-SSBs included water, no added sugar (NAS) fruit juice, milk, entirely
157 artificially-sweetened beverages (ASBs) and other non-SSBs (see Appendix Table 1).

158 We identified nutrient content for every beverage at the most detailed level possible (e.g. brand, flavour).
159 We relied on Nutribase nutrient content for international brands (and cross-checked these with local
160 nutrient information panels for consistency). For brands not included in Nutribase, we collected nutrient
161 information directly from product packaging and manufacturer websites (see Appendix Text 1).

162 ***Covariates***

163 Demographic information and education history were collected at the first visit. We dichotomized age
164 (25-44 years old, 45-64 years old) and education (secondary education or less compared to tertiary
165 education, which included undergraduate, postgraduate and technical/vocational training).

166 **Statistical Methods**

For peer review only - http://bmjopen.bmj.com/site/about/guidelines.xhtml

1: Levels of SSB consumption

We estimated the prevalence of SSB consumption, and descriptive statistics (mean ± 1.96×standard deviation) of levels of SSB consumption among consumers and the percentage of TEI from SSB-derived free sugars, stratified by covariates. Since SSB consumption was right-skewed (see Appendix Figures 1 and 2), we report volume (in 250 mL servings) and percent of TEI using geometric means and SDs. To enable comparison with global estimates, we re-estimated overall SSB intake only using the arithmetic mean, including non-consumers and using 8 oz. as a serving size.[2]

2: Percentage of SSB-derived free sugars captured by tax

We re-estimated the prevalence of SSB consumption and percentage of TEI attributable to SSB-derived free sugars separately for taxed and untaxed SSBs. Then we calculated the percentage of total SSB-derived free sugars[3] subject to the tax.

3: Free-sugar concentration

We estimated mean free-sugar concentration by SSB sub-category (i.e. separately for sodas, SSB juice drinks, etc.), weighted by reported consumption.[4] To illustrate how nutritional survey data may be used to assess potential SSB tax tiers, we report mean per-person daily volume consumed by grams of free sugar per 100mL.

All analyses were weighted by sampling probability and conducted using Stata 14.0 (StataCorp LP, Texas, United States).

For peer review only

[2] Defined by Singh et al. as 226.8 grams (equivalent to 226.8 mL), or 8 imperial ounces[25]

[3] In calculating total SSB-derived free sugars, we excluded free sugars from non-SSBs (such as free sugars in NAS juice) and sugars naturally present in milk (which are not included in the definition of free sugars)[1].

[4] Consumption-weighted estimates of free-sugar concentration were used to reflect consumption patterns (compared to reflecting the distribution of available free-sugars in the market)

For peer review only - http://bmjopen.bmj.com/site/about/guidelines.xhtml

This study is reported according to the Strengthening Reporting of Observational Studies in Epidemiology Extension for Nutritional Epidemiology (STROBE-nut) checklist (see Appendix Table 2).[36]

Results

1: Levels of SSB consumption

Eighty-eight percent of participants reported consuming SSBs at least once over the two days (Table 1). Prevalence of SSB consumption did not differ significantly between sub-groups. Amongst those who reported any consumption, mean per-person daily SSB intake was 2.4 250mL servings (mean±2 SD, 0.6,9.2). To enable comparison with published estimates, we also report mean per-person daily SSB intake in 8 oz. servings across the whole study population (2.7 8 oz. servings (95%CI 2.5, 2.9)). Men and those with less education reported consuming a higher volume of SSBs than their counterparts (p-values of <0.001 and 0.004 respectively). TEI from SSB-related free sugars was 9.2% (mean±2×SD, 2.1,41.3), with a similar patterning of results by sub-groups.

2: Percentage captured by tax

Seventy five percent of participants consumed taxed SSBs, and a similar percentage consumed untaxed SSBs (Table 2). A higher percentage of men consumed taxed SSBs as compared to women (p=0.035). TEI attributable to taxed SSBs was 6.7% (mean±2SD 1.7,26.5), and TEI attributable to untaxed SSBs was 3.5% (mean±2SD 0.4,27.3). Those with less education consumed a higher percentage of TEI from taxed SSBs than those with higher education (p=0.01). Sixty-one percent of SSB-derived free sugars were taxed (95%CI 55.7,66.5), with no significant differences by sub-group.

3: Free-sugar concentration

We estimated mean consumption-weighted free sugar concentration for each product category. As summarized in Figure 1, sweetened condensed milk was associated with the highest concentration of free

For peer review only - http://bmjopen.bmj.com/site/about/guidelines.xhtml

sugars (70 gr/100mL). Mauby, juice drinks, and sodas had the next highest average free sugar concentrations. Five of the nine beverage types with more than 6.25 grams free sugar/100 mL (Chile's SSB tax threshold) were untaxed. We also report mean per-person free sugar consumed (taking into account sugar concentration and consumption levels), by product type (see Appendix Figure 3). We assessed the mean per-person daily consumption of soft drinks (excluding those with free sugar<1 gr/100mL, and including home-prepared SSBs and no added sugar juice) by free sugar concentration (Figure 2), stratified by taxed/untaxed SSBs. Half of the drinks consumed with the highest free sugar levels (12+gr/100mL) were not subject to the tax (see Appendix Table 3 and Appendix Text 2 for examples of the products in each category by free sugar concentration).

Discussion

We used pre-existing nutritional survey data to assess three important considerations around the introduction and design of the Barbados SSB tax.

SSB consumption levels amongst adults aged 25-64 years in Barbados were very high (2.7 8-oz. servings/day, 95% CI 2.5,2.9) compared to global estimates (0.58 8-oz. servings/day, 95%CI 0.37, 0.83), suggesting that interventions to reduce SSB consumption in Barbados had the potential to reduce absolute free sugar consumption more than in settings with low baseline consumption.[25] SSB-derived free sugar accounted for 9.2% of TEI (mean±2 SD 2.1,41.3), and therefore nearly half of the population exceeded the WHO's recommendation for *total* free sugar (10%, including sweets, jams, confectionary, etc.) solely from SSB consumption.[1]

The Barbados SSB tax captured a moderate percentage of SSB-derived free sugars (61.1%, 95% CI 55.7,66.5), possibly incentivizing substitution to untaxed SSBs and dampening the potential health impact of the tax.

The Barbados SSB tax did not clearly differentiate between consumption-weighted high- and low-sugar products, which may further incentivize substitution to high-sugar untaxed alternatives in particular.

For peer review only - http://bmjopen.bmj.com/site/about/guidelines.xhtml

Strengths and Limitations

The considerations assessed here reflect some aspects of SSB tax design, but many other context-specific factors need to be considered (e.g. tax level, tax structure, availability of alternative beverages, public acceptability, market structure, revenue ear-marking, related policies, etc.). However, this assessment illustrated important aspects of context-specific consumption patterns and may provide useful information to policymakers.

Given the data available, we were not able to assess SSB consumption patterns amongst children, young adults or adults over 65. The combined response rate was 45%, comparable to that of a similar national dietary survey in the United Kingdom (47%).[37] Survey weights were used to take the population representativeness into account as much as possible and to match the age and sex distribution of the Barbados population. However, if participants differed systematically from non-participants in ways not accounted for by the survey weights, our estimates of SSB consumption may not be representative of the broader population. There was a dip in recalls conducted between July-September, suggesting that recall data may be slightly seasonally biased. July-September represent the hottest months in Barbados and SSB consumption may increase during these months, which would imply that we may have underestimated consumption.[38] Underestimation could have also occurred because of the subjectivity in the two 24-hour recall data, which may have been partially mitigated by the energy density approach (% of TEI).

In relation to other studies

The Global Burden of Disease (GBD) 2010 study estimated that SSB consumption in Barbados was between 2.0 to 2.4 8-oz servings/day, lower than our comparable estimate of 2.7 servings/day.[25] This difference may reflect that the GBD estimate for Barbados was derived from a study conducted in Jamaica between 1993-1995[39] and an unpublished analysis.[25]

In comparison to national measures of SSB consumption from other settings, our estimates were relatively high. Han & Powell estimated the two-day prevalence of SSB consumption amongst US adults

For peer review only - http://bmjopen.bmj.com/site/about/guidelines.xhtml

was 50%, lower than our comparable estimate of 89% amongst adults in Barbados.[40] A study of Dutch adults found that SSBs and non-SSBs accounted for 5.1% of TEI and a study of Australian children estimated an SSB contribution of 4.4%, much lower than our 9.2% estimate.[2,41,42]

This is the first study that we are aware of to quantify the percentage of SSB-derived free sugars covered by an SSB tax. Given heterogeneous SSB consumption worldwide, it would be valuable to repeat this approach in different settings to assess both the potential (in general) of an SSB tax to target sources of soft drink-derived free sugar, as well as to evaluate the specific definition of proposed future taxes. Powell et al. have assessed the distribution of sugar concentration by consumption of ready-to-drink SSBs (excluding home-prepared SSBs) in the US, and identified two clusters of highly-consumed concentration levels.[28] They recommended that SSB tax thresholds should be set at 5 gr/8 oz. below these highly-consumed clusters to encourage reformulation.[28] This guidance would imply a threshold of around 8gr/100mL given the distribution we observed in Barbados, somewhat higher than the threshold used in Chile (6.25gr/100mL).[43] More empirical work is needed to understand how companies respond to these thresholds in practice, and to assess how home-prepared SSBs compare in terms of sugar concentration levels in other settings.

Meaning of the study

Implications for Barbados

Adult SSB consumption levels were high before the introduction of the Barbados SSB tax. However, the definition of taxable products suggests that the tax was only likely to cover a moderate proportion of SSB-related free sugar consumption. While the Barbados tax was amended in 2017 to include store-bought mauby syrup (tariff heading 21.06), homemade mauby and other homemade SSBs remain difficult to address through a tax.[44] To maximize health benefit, the tax could be further amended to cover a higher proportion of SSB-derived free sugars, such as powdered juice drinks and powdered hot chocolate.

For peer review only - http://bmjopen.bmj.com/site/about/guidelines.xhtml

Some untaxed products (e.g. no added sugar juices, powdered juices) contain higher levels of free sugars than taxed products, suggesting that substitution to untaxed beverages could have the unintended consequence of increasing sugar consumption. To further maximize health benefit, the tax could be amended to include some of these products. For example, including no added sugar juices in the SSB tax may further help to deter free sugar consumption.[45–47] Recent dietary guidelines in Barbados suggest limiting no added sugar juice intake to 250 ml/day, and similar guidelines in the UK recommend a threshold of less than 150ml/day. In addition, different tax designs may be considered, such as basing the tax on sugar content or introducing sugar-content based tiers, as has been done elsewhere.[20]

Implications for other settings

We found that it was feasible to use pre-existing nutritional survey data to assess these considerations, and suggest that they may usefully inform SSB tax design.

In countries which use tariff headings as the basis for SSB taxation (e.g. Barbados, St. Kitts and Nevis, Bolivia and South Africa), the tariff headings selected for taxation may vary substantially.[48–50] For example, in South Africa taxable tariff headings included 18.06 ("cocoa powder… for making beverages"), 19.01 ("malt extract… for making beverages"), 21.06 ("syrups and other concentrates or preparation for making beverages") and 22.02 ("waters…containing added sugar…"), while in Barbados taxable tariff headings (in the original law) included 20.09 (" Fruit juices … and vegetable juices…") and 22.02 ("waters…containing added sugar…") and the 2017 amendment included 21.06 ("mauby syrup and other flavoured or coloured sugar syrups").[18,44,50] When SSB taxes are defined by tariff headings or other types of product categories, care should be taken that all high-sugar products are taxed to limit incentives for substitution.

A potential limitation of SSB taxes in general is that they do not cover home-prepared SSBs. In contexts where a high absolute volume of SSBs are home-prepared, an SSB tax has less health potential irrespective of the definition of taxable products. Complementary mass media or education campaigns

For peer review only - http://bmjopen.bmj.com/site/about/guidelines.xhtml

that target untaxed sources of SSB-derived free sugars may be helpful in addressing free sugar consumption overall, given the limitations of any tax to capture all of these beverages.

It was feasible to use existing nutritional survey data to assess several important considerations around SSB taxation, and these data offered some advantages over other potential data sources. Nutritional survey data can provide insight around homemade and on-the-go SSB consumption, although they may be limited by small sample sizes (which may preclude sub-group analyses) and infrequent administration. Nevertheless, standard nutritional surveys, when combined with detailed nutrient content data, can provide an opportunity to assess consumption patterns and highlight opportunities to design tailored, context-informed SSB taxes.

Conclusion

We used nutritional survey data to demonstrate high levels of SSB consumption (both in volume and as a percentage of total energy intake) amongst adults in Barbados prior to the introduction of the Barbados SSB tax. The Barbados SSB tax could be amended to apply to additional SSB products, potentially increasing possible health benefits. SSB taxes may miss home-prepared SSBs, and additional interventions may be needed to address these sources of free-sugars. Evaluating these considerations (baseline SSB consumption levels, the percentage of all SSBs that would be taxed, and the ability of a tax to differentiate between high- and low-sugar soft drinks) in other settings may help to improve SSB tax design and increase potential positive health impacts.

For peer review only

Box 1: Proposed considerations to help inform design of sugar-sweetened beverage (SSB) taxes using pre-existing nutritional survey data

Box 1: Proposed considerations to help inform design of sugar-sweetened beverage (SSB) taxes using pre-existing nutritional survey data

1. Baseline levels of SSB consumption and contribution of SSB-derived free sugar to total energy intake (TEI)
2. Percentage of SSB consumption covered by SSB tax
3. Extent to which SSB tax differentiates between high- and low-sugar SSBs

Table 1: Consumption of sugar-sweetened beverages amongst adults aged 25-64 years by demographic characteristics, Barbados 2012-2013: Barbados Salt Intake Study (n=334)

		**Distribution** (n=334)	**Prevalence of any SSB consumption**[1] (n=334)		**Volume (servings/day), given SSB consumption**[2,3] (n=300)		**TEI from SSB free sugars**, given SSB consumption**[2,5] (n=300)	
		%	%	95% CI	Mean	Mean±2 SD[4]	%	Mean±2 SD[4]
Overall	Total		88.8	85.1,92.5	2.4	0.6,9.2	9.2	2.1,41.3
By Subgroup								
Age	*25-44*	51.1	89.1	83.7,94.6	2.7	0.9,8.3	10.3	3.0,35.6
	45-64	48.9	88.4	82.1,94.7	2.2	0.5,9.8	8.2	1.5,46.6
Sex	*Males*	48.8	89.7	83.7,95.7	2.8*	0.9,9.1	10.5*	2.7,41.3
	Females	51.2	87.9	83.0,92.9	2.1*	0.5,8.6	8.2*	1.7,39.6
Education	*<Tertiary*	62.9	90.9	85.7,96.2	2.7*	0.8,9.2	10.0*	2.4,41.3
	Tertiary+	37.1	85.1	78.1,92.2	2.0*	0.5,8.4	8.0*	1.6,39.6

* Significant at p-value<0.05 in survey-weighted bivariate logistic regression (prevalence of any SSB consumption models) or survey-weighted bivariate generalized linear regression with log-link function (volume, TEI models)
[1] Defined as >0 gr of any SSB across two 24-hour recalls
[2] Geometric means
[3] Defined as the mean volume (250 mL servings/day) from SSBs, amongst all SSB-consumers. For estimates of 8 oz per serving, each value is to be multiplied by 0.91.
[4] Geometric mean±1.96 SD derived from the log-transformed variable to reflect the sample distribution
[5] Defined as the percentage of total energy intake (TEI) from SSB-derived free sugars, amongst all SSB-consumers

For peer review only - http://bmjopen.bmj.com/site/about/guidelines.xhtml

Table 2: Prevalence of consumption and total energy intake (TEI) (%) from sugar-sweetened beverage (SSB)-derived free sugars among adults aged 25 to 64 years, stratified by subsequent taxable status, Barbados 2012-2013: Barbados Salt Intake Study (n=334)[1]

		Prevalence of any SSB Consumption[2]				TEI** from SSB-derived free sugars, given any SSB consumption[3,4]				SSB-derived free sugars from taxed SSBs[6]	
		Taxed SSBs (n=334)		Untaxed SSBs (n=334)		Taxed SSBs (n=239)		Untaxed SSBs (n=249)		Percentage Taxed (n=300)	
		%	95% CI	%	95% CI	%	Mean±2 SD	%	Mean±2 SD	%	95% CI
Overall	Total	74.6	69.8,79.5	74.5	69.8,79.2	6.7	1.7,26.5	3.5	0.4,27.3	61.1	55.7,66.5
By Subgroup											
Age	*25-44*	80.8	73.7,87.9	75.0	67.5,82.6	7.0	1.9,25.7	3.6	0.6,22.6	64.2	58.3,70.1
	45-64	68.1	59.5,76.8	74.0	65.5,82.4	6.3	1.4,27.3	3.4	0.3,33.3	57.0	47.5,66.5
Sex	*Males*	79.8*	72.8,86.8	70.8	62.3,79.3	7.2	1.9,27.9	3.9	0.5,30.4	62.1	56.0,68.2
	Females	69.7*	63.1,76.3	78.0	72.9,83.2	6.1	1.5,24.6	3.2	0.4,24.1	59.6	51.5,67.7
Education	*<tertiary*	77.4	70.5,84.3	76.8	70.2,83.5	7.2*	1.8,28.6	3.5	0.5,26.9	63.2	57.7,68.7
	Tertiary+	69.9	61.8,78.0	70.6	63.5,77.7	5.7*	1.5,21.8	3.4	0.4,28.0	56.7	48.1,65.3

* Significant at p-value<0.05 in survey-weighted bivariate logistic regression (prevalence of any SSB consumption models) or bivariate generalized linear regression with log-link function (TEI)

[1] The tax was introduced in 2015, so we retrospectively apply the definition of taxable goods to consumption data reported from 2012-2013

[2]Defined as >0 gr of taxed/untaxed SSBs across two 24-hour recalls

[3] Geometric means

[4] Defined as the mean total energy intake (TEI) from SSB-derived free sugars divided by TEI, amongst all taxed and untaxed SSB-consumers separately

[5] 95% confidence interval defined as mean±1.96 SD to reflect the sample distribution

[6] Defined as the percentage of SSB-derived free sugars that were included in the original Barbados SSB tax definition of taxable products, amongst all SSB-consumers

For peer review only

Figure_1_file

Figure 1: Mean consumption-weighted free sugar concentration by product type (gr/100mL) stratified by subsequent taxable status, and mean per-person daily volume consumed (mL) in Barbados 2012-2013: the Barbados Salt Intake Study (n=334)[1, 2]

[1] We present no added sugar (NAS) juice sugars for comparison in the figure, and include a dashed line to represent the SSB tax threshold used in Chile (6.25 gr sugar/100mL) [1,43].

[2] The tax was introduced in 2015, so we retrospectively apply the definition of taxable goods to consumption data reported from 2012-2013

For peer review only

Figure_2_file

Figure 2: Mean per-person daily volume consumed (mL) by free sugar concentration (gr/100mL and gr/8 oz.), stratified by subsequent taxable status in Barbados 2012-2013: the Barbados Salt Intake Study (n=334)[1]

[1] The tax was introduced in 2015, so we retrospectively apply the definition of taxable goods to consumption data reported from 2012-2013

For peer review only

For peer review only - http://bmjopen.bmj.com/site/about/guidelines.xhtml

Acknowledgements

The full Barbados SSB Tax Evaluation Group is: Trevor Hassell (Chairperson of the group, National NCD Commission of Barbados and Healthy Caribbean Coalition); Miriam Alvarado (CEDAR, University of Cambridge); Kenneth George (Ministry of Health), Ian Hambleton (UWI); Maisha Hutton (Healthy Caribbean Coalition); Winston Moore (UWI); Madhuvanti Murphy (UWI); Alafia Samuels (UWI); Godfrey Xuereb (PAHO); Nigel Unwin (UWI; CEDAR, University of Cambridge); Cyril Gill (Ministry of Finance) and Arthur Phillips (Ministry of Health).

The group is supported by a Technical Advisory Committee: Jean Adams (CEDAR, University of Cambridge); Deliana Kostova (US Center for Disease Control and Prevention); Marc Suhrcke (University of York); Anne Marie Thow (University of Sydney)

Nita Forouhi generously offered her time to review a preliminary statistical analysis plan and Dave Collins provided technical expertise around survey-based estimates of SSB consumption.

Funding

The overall evaluation received support from the Canadian International Development Research Centre (grant no. 107604-001) and from the US Center for Disease Control and Prevention (TEPHINET). MA is funded through a Gates Cambridge PhD Scholarship, and received travel funding from Robinson College, the Global Food Security Fund, the Luca D'Agliano Scholarship, the Yates Unilever Fund and the Smuts Memorial Fund. JA is funded by the Centre for Diet and Activity Research (CEDAR), a UKCRC Public Health Research Centre of Excellence. FI was funded by the Medical Research Council Epidemiology Unit Core Support (MC_UU_12015/5). Funding from the British Heart Foundation, Cancer Research UK, Economic and Social Research Council, Medical Research Council, the National Institute for Health Research, and the Wellcome Trust, under the auspices of the UK Clinical Research Collaboration, is gratefully acknowledged. The Health of the Nation Study and the Barbados Salt Intake Study received funding from The Ministry of Health of the Government of Barbados.

The findings and conclusions in this report are those of the authors and do not necessarily represent the official position of any funding agencies.

Conflict of Interest Statement

The authors declare there is no financial conflict of interest.

Ethics Statement

Ethics approval was given by the University of the West Indies Cavehill Institutional Review Board.

Data sharing statement

The datasets analysed during the current study (deidentified participant data) are available from the corresponding author (0000-0003-2864-9410) on reasonable request.

For peer review only

359 **Contributors**

360 MA was involved in conceptualizing the study, developing the methodology, analyzing the data and
361 writing and revising the manuscript. NU was involved in conceptualization, funding acquisition,
362 developing the methodology, and reviewing and editing the manuscript. JA was involved in
363 conceptualization, developing the methodology, and reviewing and editing the manuscript. RH was
364 involved in data curation and data quality checks, developing the methodology, and reviewing and editing
365 the manuscript. AR was involved in data curation and software development, developing the
366 methodology, validating results and reviewing and editing the manuscript. IH was involved in data
367 curation, developing the methodology, reviewing data visualizations, and reviewing and editing the
368 manuscript. FI was involved in developing the methodology, reviewing data presentation, and reviewing
369 and editing the manuscript. All authors read and approved the final manuscript.

For peer review only

For peer review only - http://bmjopen.bmj.com/site/about/guidelines.xhtml

References

1 World Health Organization. Guideline: sugars intake for adults and children. Geneva: World Health Organization, 2015.

2 Sluik D, van Lee L, Engelen A, Feskens E. Total, Free, and Added Sugar Consumption and Adherence to Guidelines: The Dutch National Food Consumption Survey 2007–2010. *Nutrients* 2016; **8**: 70.

3 Keast DR, Fulgoni VL, Nicklas TA, O'Neil CE. Food Sources of Energy and Nutrients among Children in the United States: National Health and Nutrition Examination Survey 2003–2006. *Nutrients* 2013; **5**: 283–301.

4 O'Neil CE, Keast DR, Fulgoni VL, Nicklas TA. Food Sources of Energy and Nutrients among Adults in the US: NHANES 2003–2006. *Nutrients* 2012; **4**: 2097–120.

5 O'Connor L, Imamura F, Lentjes MAH, Khaw K-T, Wareham NJ, Forouhi NG. Prospective associations and population impact of sweet beverage intake and type 2 diabetes, and effects of substitutions with alternative beverages. *Diabetologia* 2015; **58**: 1474–83.

6 Imamura F, O'Connor L, Ye Z, *et al.* Consumption of sugar sweetened beverages, artificially sweetened beverages, and fruit juice and incidence of type 2 diabetes: systematic review, meta-analysis, and estimation of population attributable fraction. *BMJ* 2015; **351**: h3576.

7 Hu FB. Resolved: There is sufficient scientific evidence that decreasing sugar-sweetened beverage consumption will reduce the prevalence of obesity and obesity-related diseases. *Obes Rev Off J Int Assoc Study Obes* 2013; **14**: 606–19.

8 Chazelas E, Srour B, Desmetz E, *et al.* Sugary drink consumption and risk of cancer: results from NutriNet-Santé prospective cohort. *BMJ* 2019; **366**: l2408.

9 Fuchs MA, Sato K, Niedzwiecki D, *et al.* Sugar-Sweetened Beverage Intake and Cancer Recurrence and Survival in CALGB 89803 (Alliance). *PLOS ONE* 2014; **9**: e99816.

10 Miles FL, Neuhouser ML, Zhang Z-F. Concentrated sugars and incidence of prostate cancer in a prospective cohort. *Br J Nutr* 2018; **120**: 703–10.

11 World Health Organization. Tackling NCDs: 'Best buys' and other recommended interventions for the prevention and control of noncommunicable diseases. World Health Organization, 2017 https://apps.who.int/iris/bitstream/handle/10665/259232/WHO-NMH-NVI-17.9-eng.pdf?sequence=1&isAllowed=y (accessed Sept 12, 2019).

12 Waqanivalu T, Nederveen L, World Health Organization. Fiscal policies for diet and prevention of noncommunicable diseases: technical meeting report, 5-6 May 2015, Geneva, Switzerland. 2016 http://apps.who.int/iris/bitstream/10665/250131/1/9789241511247-eng.pdf (accessed April 20, 2017).

For peer review only - http://bmjopen.bmj.com/site/about/guidelines.xhtml

13 World Health Organization. Updated Appendix 3 of the WHO Global NCD Action Plan 2013-2020. Geneva: World Health Organization, 2017 https://www.who.int/ncds/governance/technical_annex.pdf (accessed Oct 9, 2019).

14 The Task Force on Fiscal Policy for Health. Health Taxes to Save Lives: Employing Effective Excise Taxes on Tobacco, Alcohol, and Sugary Beverages. Chairs: Michael R. Bloomberg and Lawrence H. Summers. New York: Bloomberg Philanthropies, 2019 https://www.bbhub.io/dotorg/sites/2/2019/04/Health-Taxes-to-Save-Lives.pdf (accessed June 8, 2019).

15 World Health Organization. Taxes on sugary drinks: Why do it? World Health Organization, 2016 http://apps.who.int/iris/bitstream/10665/250303/1/WHO-NMH-PND-16.5-eng.pdf (accessed May 17, 2017).

16 Smith E, Scarborough P, Rayner M, Briggs A. Should we tax unhealthy food and drink? *Proc Nutr Soc* 2018; **77**: 314–20.

17 Advocating for Sugar-Sweetened Beverage Tax. Johns Hopkins Bloom. Sch. Public Health. http://www.jhsph.edu/departments/health-behavior-and-society/public-health-practice/practice-highlights/advocating-for-sugar-sweetened-beverage-tax.html (accessed Oct 20, 2015).

18 Sinckler C. Presentation of the Financial Statement and Budgetary Proposals 2015. 2015 https://www.barbadosparliament.com/uploads/document/d1efb84aac6a7abe4c6c0efcf8ceedd2.pdf (accessed May 25, 2017).

19 Thow AM, Quested C, Juventin L, Kun R, Khan AN, Swinburn B. Taxing soft drinks in the Pacific: implementation lessons for improving health. *Health Promot Int* 2011; **26**: 55–64.

20 World Cancer Research Fund International. NOURISHING: Use economic tools to address food affordability and purchase incentives. London: World Cancer Research Fund International, 2019 https://www.wcrf.org/sites/default/files/Use-economic-tools.pdf (accessed Nov 9, 2018).

21 Chaloupka FJ, Powell LM, Warner KE. The Use of Excise Taxes to Reduce Tobacco, Alcohol, and Sugary Beverage Consumption. *Annu Rev Public Health* 2019; **40**: null.

22 Tedstone A, Targett V, Allen R, Staff at PHE. Sugar Reduction: The evidence for action. London, UK: Public Health England, 2015 https://assets.publishing.service.gov.uk/government/uploads/system/uploads/attachment_data/file/470179/Sugar_reduction_The_evidence_for_action.pdf (accessed Dec 17, 2018).

23 Khatibzadeh S, Saheb Kashaf M, Micha R, *et al.* A global database of food and nutrient consumption. *Bull World Health Organ* 2016; **94**: 931–4.

24 Micha R, Coates J, Leclercq C, Charrondiere UR, Mozaffarian D. Global Dietary Surveillance: Data Gaps and Challenges. *Food Nutr Bull* 2018; **39**: 175–205.

For peer review only - http://bmjopen.bmj.com/site/about/guidelines.xhtml

25 Singh GM, Micha R, Khatibzadeh S, *et al.* Global, Regional, and National Consumption of Sugar-Sweetened Beverages, Fruit Juices, and Milk: A Systematic Assessment of Beverage Intake in 187 Countries. *PLOS ONE* 2015; **10**: e0124845.

26 Jou J, Techakehakij W. International application of sugar-sweetened beverage (SSB) taxation in obesity reduction: Factors that may influence policy effectiveness in country-specific contexts. *Health Policy* 2012; **107**: 83–90.

27 Adam AS, Smed S. The effects of different types of taxes on soft-drink consumption. *Inst Food Resour Econ* 2012; : 43.

28 Powell LM, Andreyeva T, Isgor Z. Distribution of sugar-sweetened beverage sales volume by sugar content in the United States: implications for tiered taxation and tax revenue. *J Public Health Policy* 2020; published online Jan 22. DOI:10.1057/s41271-019-00217-x.

29 Caribbean Community Secretariat. Revised Common External Tariff of the Caribbean Community. 2018 https://caricom.org/documents/16273-revised_cet_of_caricom_hs_2017_revised_11_april_2018_(for_link).pdf (accessed Oct 8, 2019).

30 Central Intelligence Agency. Central America: Barbados. World Factb. https://www.cia.gov/librarY/publications/the-world-factbook/geos/bb.html (accessed Nov 23, 2018).

31 Howitt C, Hambleton IR, Rose AMC, *et al.* Social distribution of diabetes, hypertension and related risk factors in Barbados: a cross-sectional study. *BMJ Open* 2015; **5**: e008869.

32 Unwin N, Rose A, George K, Hambleton I, Howitt C. The Barbados Health of the Nation Survey: Core Findings. St Michael, Barbados: Chronic Disease Research Centre, The University of the West Indies and the Barbados Ministry of Health, 2015 http://www.archive.healthycaribbean.org/newsletters/aug-2015/CDRC_HealthOfTheNationSurvey.pdf (accessed April 14, 2020).

33 Harris RM, Rose AMC, Hambleton IR, *et al.* Sodium and potassium excretion in an adult Caribbean population of African descent with a high burden of cardiovascular disease. *BMC Public Health* 2018; **18**. DOI:10.1186/s12889-018-5694-0.

34 Raper N, Perloff B, Ingwersen L, Steinfeldt L, Anand J. An overview of USDA's Dietary Intake Data System. *J Food Compos Anal* 2004; **17**: 545–55.

35 Nutribase Pro. E. Muirwood Drive, Phoenix, Arizona, USA: Cybersoft Inc., 2016.

36 Lachat C, Hawwash D, Ocké MC, *et al.* Strengthening the Reporting of Observational Studies in Epidemiology—Nutritional Epidemiology (STROBE-nut): An Extension of the STROBE Statement. *PLOS Med* 2016; **13**: e1002036.

For peer review only - http://bmjopen.bmj.com/site/about/guidelines.xhtml

37 Chambers S, Barton KL, Albani V, Anderson AS, Wrieden WL. Identifying dietary differences between Scotland and England: a rapid review of the literature. *Public Health Nutr* 2017; **20**: 2459–77.

38 World Bank. World Bank Climate Change Knowledge Portal: Barbados. https://climateknowledgeportal.worldbank.org/country/barbados (accessed Oct 9, 2019).

39 Jackson M, Walker S, Forrester T, Cruickshank JK, Wilks R. Social and dietary determinants of body mass index of adult Jamaicans of African origin. *Eur J Clin Nutr* 2003; **57**: 621–7.

40 Han E, Powell LM. Consumption patterns of sugar-sweetened beverages in the United States. *J Acad Nutr Diet* 2013; **113**: 43–53.

41 Newens KJ, Walton J. A review of sugar consumption from nationally representative dietary surveys across the world. *J Hum Nutr Diet Off J Br Diet Assoc* 2016; **29**: 225–40.

42 Louie JCY, Moshtaghian H, Rangan AM, Flood VM, Gill TP. Intake and sources of added sugars among Australian children and adolescents. *Eur J Nutr* 2016; **55**: 2347–55.

43 Caro JC, Corvalán C, Reyes M, Silva A, Popkin B, Taillie LS. Chile's 2014 sugar-sweetened beverage tax and changes in prices and purchases of sugar-sweetened beverages: An observational study in an urban environment. *PLOS Med* 2018; **15**: e1002597.

44 Government of Barbados. Excise Tax (Amendment) Regulations, 2017. 2017 https://www.barbadosparliament.com/uploads/sittings/attachments/52aa3985de8d4ef6f99b91d346914ba5.pdf (accessed Jan 20, 2020).

45 Chaloupka F, Powell L. Using Fiscal Policy to Promote Health: Taxing Tobacco, Alcohol, and Sugary Beverages. *Backgr Pap Task Force Fisc Policy Health* 2018; : 25.

46 Public Health England. The Eatwell Guide. 2018.

47 Ministry of Health. Food Based Dietary Guidelines for Barbados. 2017 http://www.fao.org/3/I9680EN/i9680en.pdf (accessed Jan 11, 2019).

48 Government of Saint Christopher and Nevis. Excise Tax Act. *Off Gaz* 2010; : 52.

49 Government of Bolivia L. Actualización de las alícuotas específicas del impuesto a los consumos específicos (ICE) para la gestión. 2018; : 5.

50 South African Revenue Service (SARS). Schedules to the Customs and Excise Act, 1964 (Tariff Book). https://www.sars.gov.za/AllDocs/LegalDoclib/SCEA1964/LAPD-LPrim-Tariff-2012-13b%20-%20Schedule%20No%201%20Part%207A.pdf (accessed Oct 23, 2019).

For peer review only

For peer review only - http://bmjopen.bmj.com/site/about/guidelines.xhtml

For peer review only

For peer review only - http://bmjopen.bmj.com/site/about/guidelines.xhtml

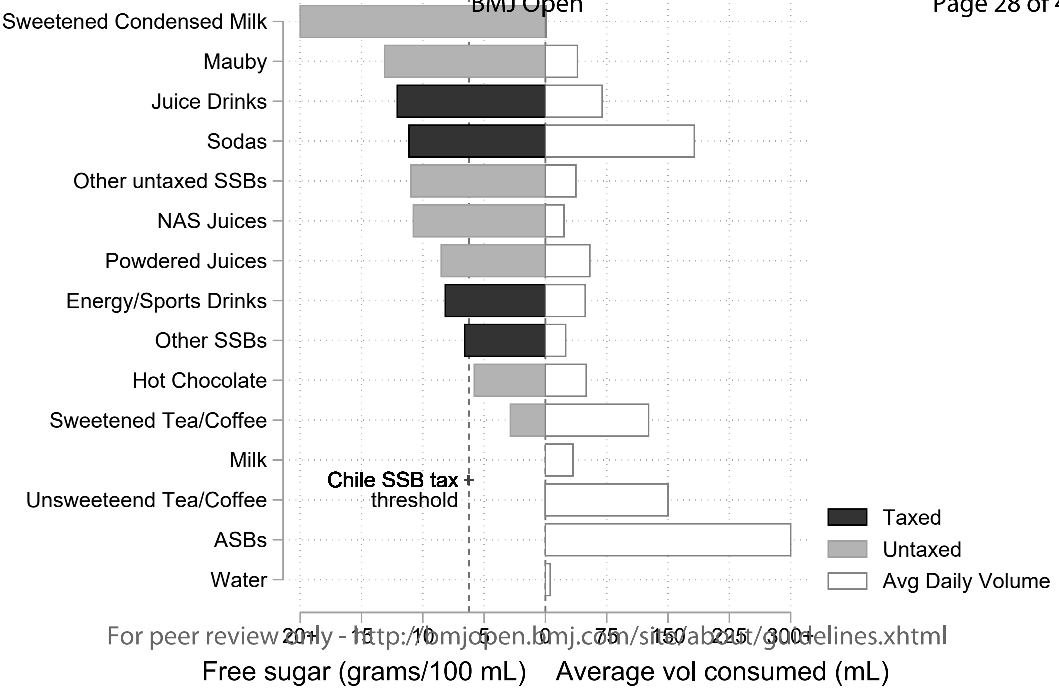

For peer review only - http://bmjopen.bmj.com/site/about/guidelines.xhtml

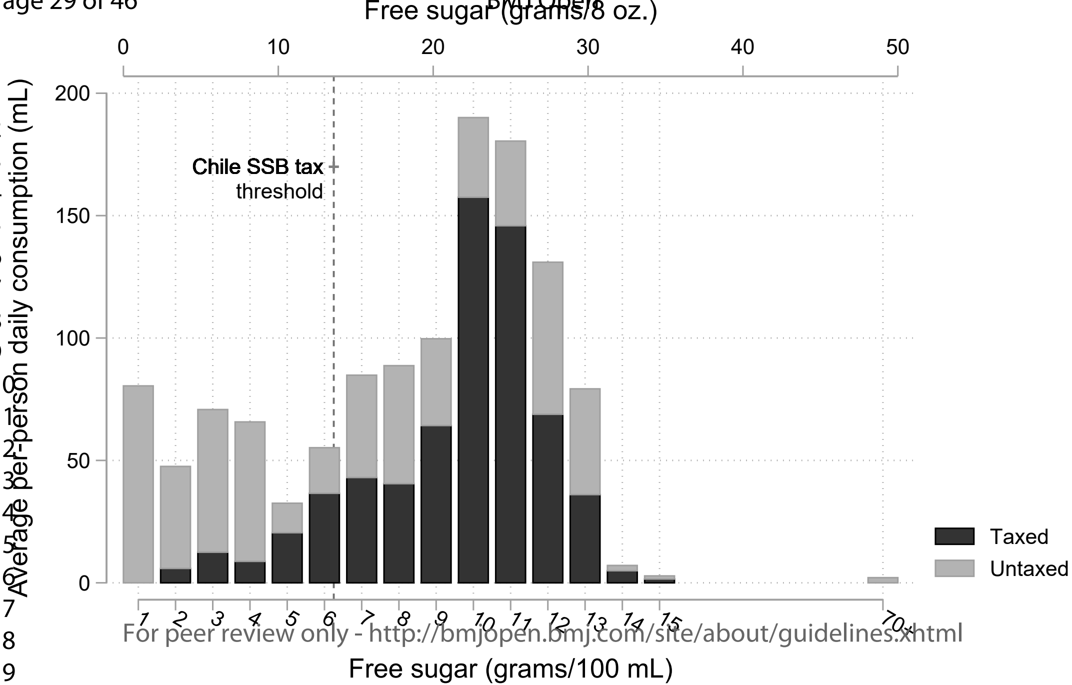

For peer review only - http://bmjopen.bmj.com/site/about/guidelines.xhtml

Appendix Files:

1. **Appendix Table 1:** Beverage category definitions
2. **Appendix Table 2:** STROBE-nut: An extension of the STROBE statement for nutritional epidemiology
3. **Appendix Table 3:** Products by taxable status and free sugar concentration levels (with specific brands as exemplars)
4. **Appendix Text 1:** Definition of beverage categories and nutrient composition
5. **Appendix Text 2:** Sugar concentration by product types
6. **Appendix Figure 1:** Distribution of SSB consumption in mean daily servings/capita, given any SSB consumption, Barbados 2012-2013: the Barbados Salt Intake Study
7. **Appendix Figure 2**: Per-person mean Total Energy Intake (TEI) attributable to SSB-related free sugars consumption (%), given any consumption, Barbados 2012-2013: the Barbados Salt Intake Study
8. **Appendix Figure 3:** Mean per-person free sugar consumed from soft drinks amongst adults aged 25-64, by product type and taxable status, Barbados 2012-2013: the Barbados Salt Intake Study

For peer review only

For peer review only - http://bmjopen.bmj.com/site/about/guidelines.xhtml

Appendix Table 1: Beverage category definitions

Main Categories	Taxable status	Level I Category	Level II Categories	Examples of frequently consumed drinks
SSBs	Taxed SSBs	Soda	Soda	Coca Cola, Frutee, Fanta, Sprite, Pepsi
		Juice Drinks	Juice Drinks	Pinehill Dairy Juice drinks, Fruta
		Energy/Sports/Malt Drinks	Malt Drinks	VitaMalt
			Energy Drinks	Redbull, Monster
			Sports Drinks	Gatorade, Lucozade
		Other taxed SSBs	Flavored SSB water	Cranwater
			Flavoured Dairy	Indulgence Milk
			Other taxed SSBs	Store-bought iced tea, Ensure, Supligen, seamoss
	Untaxed SSBs	Mauby*	Mauby*	Mauby*
		Powdered Juice Drinks	Powdered Juice Drinks	Tang, turbo, koolaid
		Hot Chocolate	Powdered hot chocolate	Milo, Nestle
		Sweetened tea/coffee	Homemade sweetened tea/coffee	coffee, tea or iced tea w/ added sugar or sweetened condensed milk
		Sweetened condensed milk	Sweetened condensed milk	Sweetened condensed milk consumed with cereal or cream of wheat as a milk substitute
		Other untaxed SSBs	Homemade SSBs juice drinks	sugar-sweetened homemade smoothies
				sugar-sweetened homemade juices
			Other untaxed SSBs	snowcone, milkshake, purchased sweetened coffee drinks (lattes, mochas etc)
Non-SSBs	Untaxed non-SSBs	Water	Water	Tap water, bottled water, soda water
		NAS juice (no added sugar)	NAS Juice	NAS Pinehill Dairy, Dewlands, Ceres
			Homemade non-SSBs	no-sugar added homemade juices
				no-sugar added homemade smoothies
		Other non-SSB	no added sugar coffee/tea	Coffee/tea with no added sugar
		Milk	Milk	Milk
		ASB	Diet Soda	Diet Coke

9. *Although mauby could be considered a homemade drink it can also be purchased as a syrup or a ready-made drink, and as such we report it as a separate category under SSBs.

For peer review only

For peer review only - http://bmjopen.bmj.com/site/about/guidelines.xhtml

Appendix Table 2: STROBE-nut: An extension of the STROBE statement for nutritional epidemiology (Lachat C et al., 2016)

Item	Item nr	STROBE recommendations	Extension for Nutritional Epidemiology studies (STROBE-nut)	Reported on page #
**Title and abstract**	1	(a) Indicate the study's design with a commonly used term in the title or the abstract.<br><br>(b) Provide in the abstract an informative and balanced summary of what was done and what was found.	**nut-1**State the dietary/nutritional assessment method(s) used in the title, abstract, or keywords.	**Abstract**
**Introduction**				
Background rationale	2	Explain the scientific background and rationale for the investigation being reported.		1-2
Objectives	3	State specific objectives, including any pre-specified hypotheses.		3
**Methods**				
Study design	4	Present key elements of study design early in the paper.		3
Settings	5	Describe the setting, locations, and relevant dates, including periods of recruitment, exposure, follow-up, and data collection.	**nut-5**Describe any characteristics of the study settings that might affect the dietary intake or nutritional status of the participants, if applicable.	3
Participants	6	a) Cohort study—Give the eligibility criteria, and the sources and methods of selection of participants. Describe methods of follow-up.<br><br>Case-control study—Give the eligibility criteria, and the sources and methods of case ascertainment and control selection. Give the rationale for the choice of cases and controls.<br><br>Cross-sectional study—Give the eligibility criteria, and the sources and methods of selection of participants.<br><br>(b) Cohort study—For matched studies, give matching criteria and number of exposed and unexposed.	**nut-6** Report particular dietary, physiological or nutritional characteristics that were considered when selecting the target population.	3-4

For peer review only - http://bmjopen.bmj.com/site/about/guidelines.xhtml

Item	Item nr	STROBE recommendations	Extension for Nutritional Epidemiology studies (STROBE-nut)	Reported on page #
		Case-control study—For matched studies, give matching criteria and the number of controls per case.		
Variables	7	Clearly define all outcomes, exposures, predictors, potential confounders, and effect modifiers. Give diagnostic criteria, if applicable.	**nut-7.1**Clearly define foods, food groups, nutrients, or other food components.	**4-5**
			**nut-7.2** When using dietary patterns or indices, describe the methods to obtain them and their nutritional properties.	**3**
Data sources - measurements	8	For each variable of interest, give sources of data and details of methods of assessment (measurement).Describe comparability of assessment methods if there is more than one group.	**nut-8.1** Describe the dietary assessment method(s), e.g., portion size estimation, number of days and items recorded, how it was developed and administered, and how quality was assured. Report if and how supplement intake was assessed.	**3-4**
			**nut-8.2** Describe and justify food composition data used. Explain the procedure to match food composition with consumption data. Describe the use of conversion factors, if applicable.	**3-4**
			**nut-8.3** Describe the nutrient requirements, recommendations, or dietary guidelines and the evaluation approach usedto compare intake with the dietary reference values, if applicable.	**5**
			**nut-8.4** When using nutritional biomarkers, additionally use the STROBE Extension for Molecular Epidemiology (STROBE-ME). Report the type of biomarkers used and their usefulness as dietary exposure markers.	**NA**
			**nut-8.5** Describe the assessment of nondietary data (e.g., nutritional status and influencing factors) and timing of the assessment of these variables in relation to dietary assessment.	**4-5**

For peer review only - http://bmjopen.bmj.com/site/about/guidelines.xhtml

Item	Item nr	STROBE recommendations	Extension for Nutritional Epidemiology studies (STROBE-nut)	Reported on page #
			**nut-8.6** Report on the validity of the dietary or nutritional assessment methods and any internal or external validation used in the study, if applicable.	**3-4**
Bias	9	Describe any efforts to address potential sources of bias.	**nut-9** Report how bias in dietary or nutritional assessment was addressed, e.g., misreporting, changes in habits as a result of being measured, or data imputation from other sources	**3-4 and Appendix Text 1-2**
Study Size	10	Explain how the study size was arrived at.		**3-4**
Quantitative variables	11	Explain how quantitative variables were handled in the analyses. If applicable, describe which groupings were chosen and why.	**nut-11** Explain categorization of dietary/nutritional data (e.g., use of N-tiles and handling of nonconsumers) and the choice of reference category, if applicable.	**4**
Statistical Methods	12	(a) Describe all statistical methods, including those used to control for confounding<br><br>(b) Describe any methods used to examine subgroups and interactions.<br><br>(c) Explain how missing data were addressed.<br><br>(d) Cohort study—If applicable, explain how loss to follow-up was addressed.<br><br>Case-control study—If applicable, explain how matching of cases and controls was addressed.<br><br>Cross-sectional study—If applicable, describe analytical methods taking account of sampling strategy.<br><br>(e) Describe any sensitivity analyses.	**nut-12.1** Describe any statistical method used to combine dietary or nutritional data, if applicable.<br><br>**nut-12.2** Describe and justify the method for energy adjustments, intake modeling, and use of weighting factors, if applicable.<br><br>**nut-12.3** Report any adjustments for measurement error, i.e,. from a validity or calibration study.	**3, 4**<br><br>**3,4, 6**
**Results**				
Participants	13	(a) Report the numbers of individuals at each stage of the study—e.g., numbers potentially eligible, examined for eligibility, confirmed eligible, included	**nut-13** Report the number of individuals excluded based on missing, incomplete or implausible dietary/nutritional data.	**4**

For peer review only - http://bmjopen.bmj.com/site/about/guidelines.xhtml

Item	Item nr	STROBE recommendations	Extension for Nutritional Epidemiology studies (STROBE-nut)	Reported on page #
		in the study, completing follow-up, and analyzed.<br><br>(b) Give reasons for non-participation at each stage.<br><br>(c) Consider use of a flow diagram.		
Descriptive data	14	(a) Give characteristics of study participants (e.g., demographic, clinical, social) and information on exposures and potential confounders<br><br>(b) Indicate the number of participants with missing data for each variable of interest<br><br>(c) Cohort study—Summarize follow-up time (e.g., average and total amount)	**nut-14** Give the distribution of participant characteristics across the exposure variables if applicable. Specify if food consumption of total population or consumers only were used to obtain results.	**Table 1, p5**
Outcome data	15	Cohort study—Report numbers of outcome events or summary measures over time.<br><br>Case-control study—Report numbers in each exposure category, or summary measures of exposure.<br><br>Cross-sectional study—Report numbers of outcome events or summary measures.		
Main results	16	(a) Give unadjusted estimates and, if applicable, confounder-adjusted estimates and their precision (e.g., 95% confidence interval).<br><br>Make clear which confounders were adjusted for and why they were included.<br><br>(b) Report category boundaries when continuous variables were categorized.<br><br>(c) If relevant, consider translating estimates of relative risk into absolute risk for a meaningful time period.	**nut-16** Specify if nutrient intakes are reported with or without inclusion of dietary supplement intake, if applicable.	**NA**
Other analyses	17	Report other analyses done—e.g., analyses of subgroups and interactions and sensitivity analyses.	**nut-17**Report any sensitivity analysis (e.g., exclusion of misreporters or	**NA**

For peer review only - http://bmjopen.bmj.com/site/about/guidelines.xhtml

Item	Item nr	STROBE recommendations	Extension for Nutritional Epidemiology studies (STROBE-nut)	Reported on page #
			outliers) and data imputation, if applicable.	
**Discussion**				
Key results	18	Summarize key results with reference to study objectives.		7
Limitation	19	Discuss limitations of the study, taking into account sources of potential bias or imprecision. Discuss both direction and magnitude of any potential bias.	**nut-19** Describe the main limitations of the data sources and assessment methods used and implications for the interpretation of the findings.	**8**
Interpretation	20	Give a cautious overall interpretation of results considering objectives, limitations, multiplicity of analyses, results from similar studies, and other relevant evidence.	**nut-20** Report the nutritional relevance of the findings, given the complexity of diet or nutrition as an exposure.	**8-10**
Generalizability	21	Discuss the generalizability (external validity) of the study results.		10
**Other information**				
Funding	22	Give the source of funding and the role of the funders for the present study and, if applicable, for the original study on which the present article is based.		**16**
Ethics			**nut-22.1**Describe the procedure for consent and study approval from ethics committee(s).	**16**
Supplementary material			**nut-22.2** Provide data collection tools and data as online material or explain how they can be accessed.	**Appendix File**

For peer review only - http://bmjopen.bmj.com/site/about/guidelines.xhtml

Appendix Table 3: Products by taxable status and free sugar concentration levels (with specific brands as exemplars)

Free sugar concentration	Taxable status	Products
**1-2.9**	Taxed	NA
	Untaxed	SSB coffee & tea, soymilk
**3-4.9**	Taxed	Flavoured water (Cranwater)
	Untaxed	Hot chocolate, soy milk, SSB coffee & tea, powdered juice (Mak-C)
**5-6.9**	Taxed	Sports drinks (Powerade), soda (Frutee - ginger ale flavor), flavored milk
	Untaxed	Powdered juice (Tang), SSB coffee & tea, powdered milk
**7-.8.9**	Taxed	Malt, Energy drinks (Plus), juice drinks
	Untaxed	Powdered juice (Turbo), SSB coffee & tea, iced tea
**9-10.9**	Taxed	Soda (Sprite, Busta), flavored milk
	Untaxed	Powdered juice (Koolaid), homemade sweet juice, NAS juice
**11.-12.9**	Taxed	Soda (Coca Cola, Frutee), juice drinks (Pinehill Dairy, Fruta)
	Untaxed	Lemonade, NAS juice, homemade juices/shakes/punch
**13-14.9**	Taxed	Juice drinks (Pinehill Dairy, Fruta), soda (Frutee, Ju-C)
	Untaxed	Mauby, homemade sweetened juice, NAS juice (Pinehill Dairy), SSB coffee & tea
**15-16.9**	Taxed	Soda, juice drinks
	Untaxed	SSB coffee & tea
**70+**	Taxed	NA
	Untaxed	Sweetened condensed milk

For peer review only

Appendix Text 1: Definition of beverage categories and nutrient composition

We categorized drinks according to the categories summarized in Appendix Table 2.

Nutribase includes nutrient information from the United States Department of Agriculture (USDA) and Canadian food composition databases. For products not included in Nutribase (e.g. local and regional brands of sodas, juices; internationally produced beverages imported from South Africa, Turkey, etc) we assigned nutrient information (sugars in grams and total calories in kilocalories) based on nutrient label data collected from product packages in stores and from websites. When a specific brand and flavour were reported in the dietary recall, we used nutrient information from the corresponding product. When no flavour was reported, we used the mean nutrient values across a range of available flavours. We relied on Nutribase nutrient information for available international brands (e.g. Coke, Sprite, etc). When no brand was reported in the dietary recall, we used the mean nutrient information for that beverage category.

For powdered drinks (powdered juices and hot chocolate) we used packet instructions to estimate reconstituted levels. Most powdered drinks reported in the recalls already include sugar and do not require additional sugar to be added. While people may add additional sugar, this was not included as a prompt in the standard 24-hr dietary recall, so our estimates of sugar intake from powdered drinks may be an underestimate.

For powdered milk we assumed a 1:5 dilution ratio and corrected levels of total calories and sugars accordingly (since the product was previously entered as undiluted powdered milk in Nutribase).

For homemade SSBs, a previous Barbados-based study used the weighed recipe approach to estimate nutrient content for three popular drinks: mauby, ginger beer and lemonade [51]. For other homemade drinks, we used the recipes that participants reported to identify similar products within Nutribase. Participants had been prompted for recipes and we used the ingredients to identify similar products within Nutribase. For homemade SSBs (smoothies and juice drinks), we categorized these as "fruit punch drink "pina colada," "blended smoothie, banana, oats, milk, honey, yogurt," "flavored milks," "pina colada," "blended shake, milkshake vanilla," "mixed berry fruit smoothie," "fruit 'n' yogurt smoothie, strawberry kiwi," "tropical fruit smoothie," "golden apple juice," "lemonade," "juice apple & cherry juice," "island guava drink," "orange flavor drink" "passion fruit juice," "dock, boiled (sorrel)," "mixed fruit juice," or "grape juice" as appropriate.

For homemade non-SSB (no added sugar smoothies and juices), we categorized these as "blended carrot, beet, celery, cucumber, apple juice without sugar," "cranberry juice," "carrot juice," "V8 60% vegetable juice, V-Lite," "aloe vera juice, " "mango juice," "orange juice, unsweetened," "lemon juice, raw," "passion fruit juice, raw," "soy milk," or "mandarin papaya drink" as appropriate.

Mauby is a local bark that is boiled with water and sugar to make a sweet drink (and can also be bought as a ready-made syrup and diluted at home or purchased ready-to-drink).

Sorrel is a flower (similar to hibiscus) that is used to make a sweetened drink. Golden apples are a fruit that are used to make a juice (often sweetened).

We excluded snowcones, as we considered these to be a dessert and not a drink.

Several drinks were categorized within Nutribase as "Pina coladas" although upon review these were identified to be homemade punches or smoothies. The sugar and total calories content of these four observation were rescaled, with pineapple punch and coconut punch re-scaled based on "fruit punch

For peer review only - http://bmjopen.bmj.com/site/about/guidelines.xhtml

drink" and "smoothie homemade" and "mango shake, homemade blended almond milk" re-scaled based on ("blended smoothie - banana oats milk honey yogurt").

To exclude galactose and lactose sugars, we subtracted these from total sugars. Where Nutribase did not automatically assign lactose/galactose sugar content to milk products, we assumed all sugars were from lactose/galactose in no added sugar milk products.

When sweetened condensed milk was reported with coffee/tea, we estimated the total sugar concentration per quantity of coffee/tea consumed and reported this under "sweetened tea/coffee" rather than "sweetened condensed milk." When consumption was reported with cereal or cream of wheat in place of regular milk we reported this under "sweetened condensed milk."

Throughout this report, "SSBs" refer to both taxed and untaxed SSBs (excluding non-SSBs), while "soft drinks" refer to both SSBs and non-SSBs. Some non-SSBs (such as no added sugar juice) contain free sugars. To clarify when non-SSBs are included, we refer to "soft-drinks" rather than "SSBs."

For participants with more than two recalls (n=1) we used only the first two recalls, assuming that reporting quality may have changed with repeated exposure to the survey instrument.

We converted all reported beverage volumes into milliliters.

Appendix Text 2: Sugar concentration by product types

The sugar concentration of some product types varied greatly, such as for home-prepared SSB tea/coffee with reported consumption at almost every sugar concentration level. Other product types were more narrowly defined (such as flavoured water, which was only found in the 3-4.9 gr/100mL category). Most of the sweetest products (13+ gr/100mL) were locally or regionally produced fruit drinks or sodas. Some flavours of no added sugar juice (non-SSBs) had a higher sugar concentration than juice drinks (SSBs), and some flavours of sodas had notably lower levels of sugar concentration than other flavours under the same brand. Sweetened condensed milk consumed as a milk substitute with cereal or cream of wheat was the only product with a sugar concentration greater than 17+ gr/100mL.

For peer review only

Appendix Figure 1: Distribution of SSB consumption in mean daily servings/capita, given any SSB consumption, Barbados 2012-2013: the Barbados Salt Intake Study

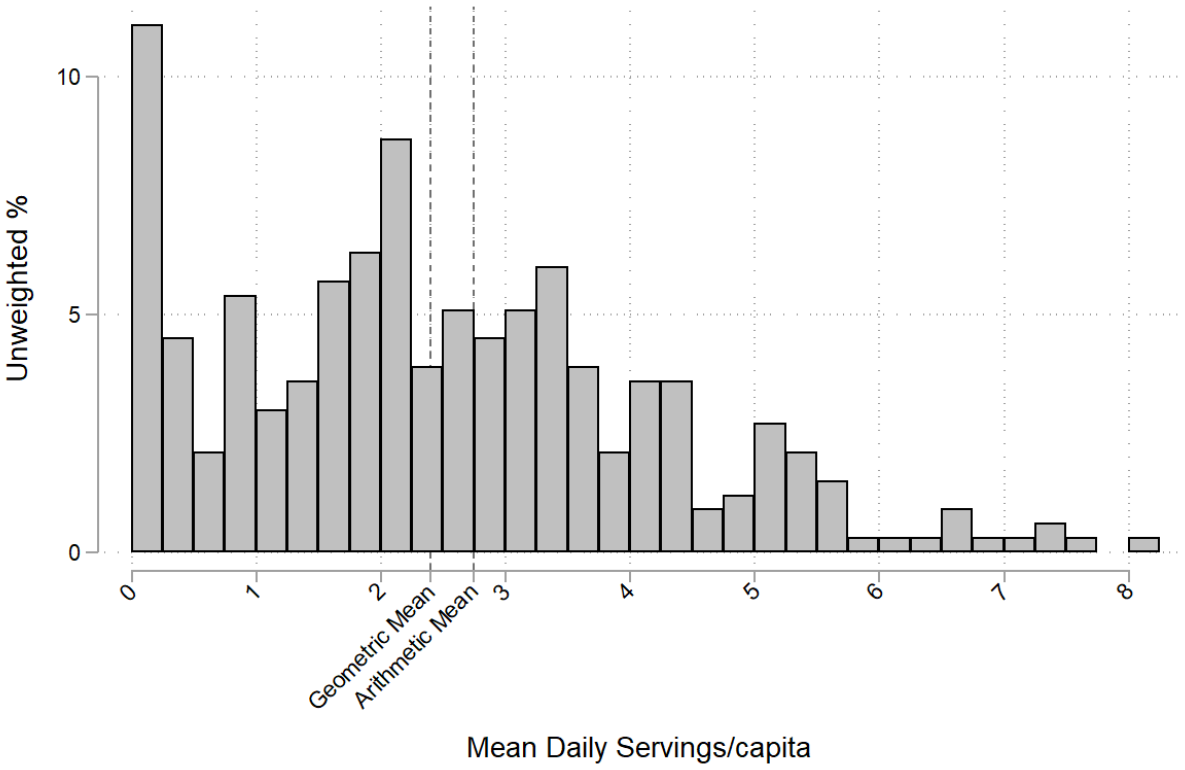

For peer review only - http://bmjopen.bmj.com/site/about/guidelines.xhtml

Appendix Figure 2: Per-person mean Total Energy Intake (TEI) attributable to SSB-related free sugars consumption (%), given any consumption, Barbados 2012-2013: the Barbados Salt Intake Study

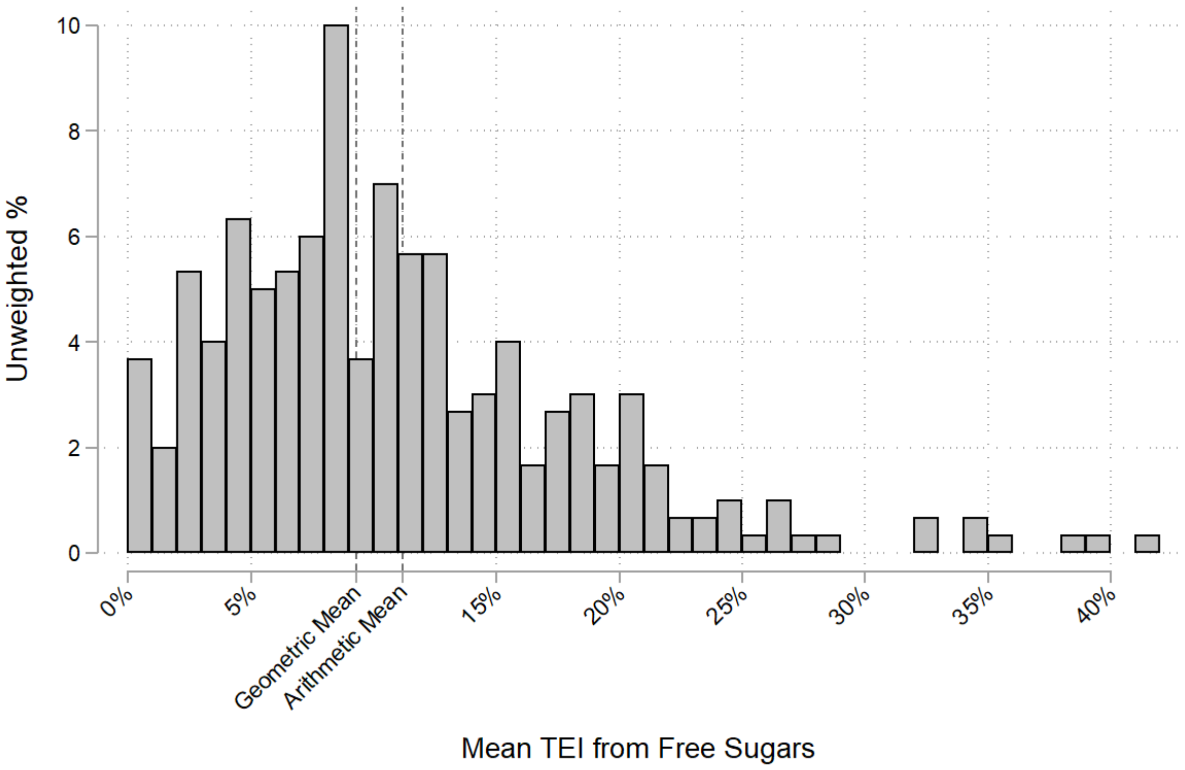

For peer review only - http://bmjopen.bmj.com/site/about/guidelines.xhtml

Appendix Figure 3: Mean per-person free sugar consumed from soft drinks amongst adults aged 25-64, by product type and taxable status, Barbados 2012-2013: the Barbados Salt Intake Study

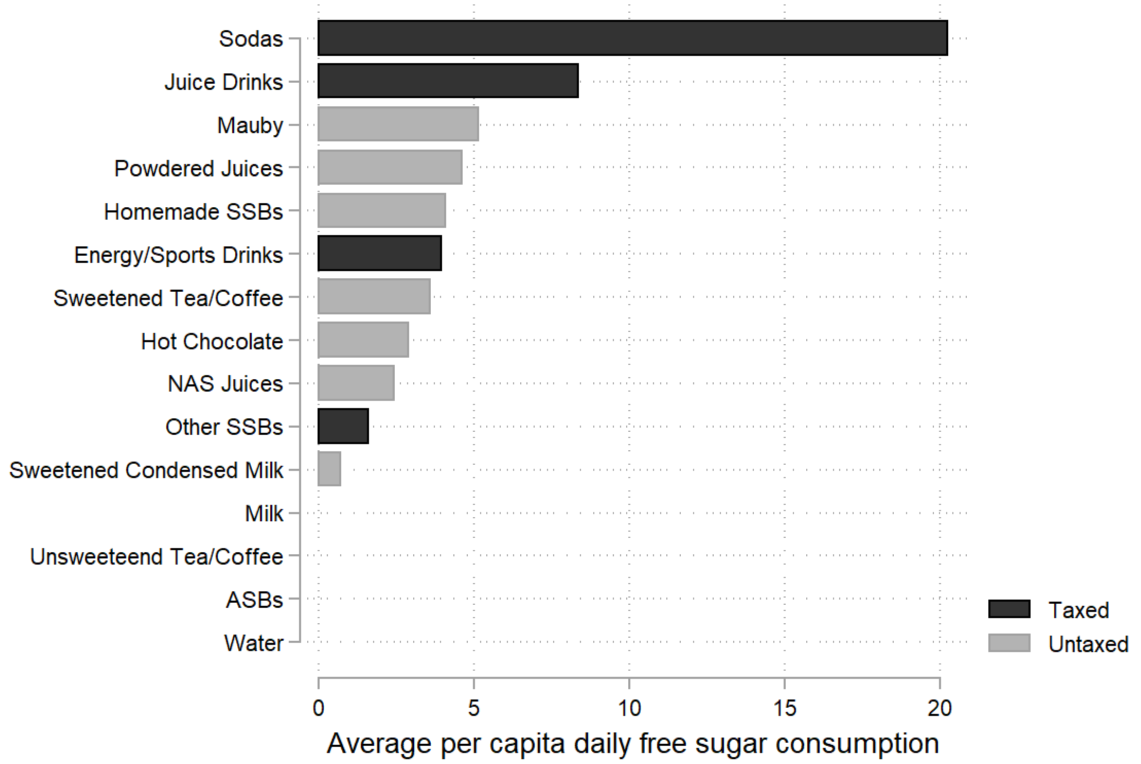

For peer review only - http://bmjopen.bmj.com/site/about/guidelines.xhtml

Research Checklist: STROBE-nut: An extension of the STROBE statement for nutritional epidemiology (Lachat C et al., 2016)

Item	Item nr	STROBE recommendations	Extension for Nutritional Epidemiology studies (STROBE-nut)	Reported on page #
**Title and abstract**	1	(a) Indicate the study's design with a commonly used term in the title or the abstract.<br><br>(b) Provide in the abstract an informative and balanced summary of what was done and what was found.	**nut-1**State the dietary/nutritional assessment method(s) used in the title, abstract, or keywords.	**Abstract**
**Introduction**				
Background rationale	2	Explain the scientific background and rationale for the investigation being reported.		1-2
Objectives	3	State specific objectives, including any pre-specified hypotheses.		3
**Methods**				
Study design	4	Present key elements of study design early in the paper.		3
Settings	5	Describe the setting, locations, and relevant dates, including periods of recruitment, exposure, follow-up, and data collection.	**nut-5**Describe any characteristics of the study settings that might affect the dietary intake or nutritional status of the participants, if applicable.	3
Participants	6	a) Cohort study—Give the eligibility criteria, and the sources and methods of selection of participants. Describe methods of follow-up.<br><br>Case-control study—Give the eligibility criteria, and the sources and methods of case ascertainment and control selection. Give the rationale for the choice of cases and controls.<br><br>Cross-sectional study—Give the eligibility criteria, and the sources and methods of selection of participants.<br><br>(b) Cohort study—For matched studies, give matching criteria and number of exposed and unexposed.	**nut-6** Report particular dietary, physiological or nutritional characteristics that were considered when selecting the target population.	3-4

For peer review only

Item	Item nr	STROBE recommendations	Extension for Nutritional Epidemiology studies (STROBE-nut)	Reported on page #
		Case-control study—For matched studies, give matching criteria and the number of controls per case.		
Variables	7	Clearly define all outcomes, exposures, predictors, potential confounders, and effect modifiers. Give diagnostic criteria, if applicable.	**nut-7.1**Clearly define foods, food groups, nutrients, or other food components.	**4-5**
			**nut-7.2** When using dietary patterns or indices, describe the methods to obtain them and their nutritional properties.	**3**
Data sources - measurements	8	For each variable of interest, give sources of data and details of methods of assessment (measurement).Describe comparability of assessment methods if there is more than one group.	**nut-8.1** Describe the dietary assessment method(s), e.g., portion size estimation, number of days and items recorded, how it was developed and administered, and how quality was assured. Report if and how supplement intake was assessed.	**3-4**
			**nut-8.2** Describe and justify food composition data used. Explain the procedure to match food composition with consumption data. Describe the use of conversion factors, if applicable.	**3-4**
			**nut-8.3** Describe the nutrient requirements, recommendations, or dietary guidelines and the evaluation approach usedto compare intake with the dietary reference values, if applicable.	**5**
			**nut-8.4** When using nutritional biomarkers, additionally use the STROBE Extension for Molecular Epidemiology (STROBE-ME). Report the type of biomarkers used and their usefulness as dietary exposure markers.	**NA**
			**nut-8.5** Describe the assessment of nondietary data (e.g., nutritional status and influencing factors) and timing of the assessment of these variables in relation to dietary assessment.	**4-5**

For peer review only

Item	Item nr	STROBE recommendations	Extension for Nutritional Epidemiology studies (STROBE-nut)	Reported on page #
			**nut-8.6** Report on the validity of the dietary or nutritional assessment methods and any internal or external validation used in the study, if applicable.	**3-4**
Bias	9	Describe any efforts to address potential sources of bias.	**nut-9** Report how bias in dietary or nutritional assessment was addressed, e.g., misreporting, changes in habits as a result of being measured, or data imputation from other sources	**3-4 and Appendix Text 1-2**
Study Size	10	Explain how the study size was arrived at.		**3-4**
Quantitative variables	11	Explain how quantitative variables were handled in the analyses. If applicable, describe which groupings were chosen and why.	**nut-11** Explain categorization of dietary/nutritional data (e.g., use of N-tiles and handling of nonconsumers) and the choice of reference category, if applicable.	**4**
Statistical Methods	12	(a) Describe all statistical methods, including those used to control for confounding	**nut-12.1** Describe any statistical method used to combine dietary or nutritional data, if applicable.	**3, 4**
		(b) Describe any methods used to examine subgroups and interactions.	**nut-12.2** Describe and justify the method for energy adjustments, intake modeling, and use of weighting factors, if applicable.	**3,4, 6**
		(c) Explain how missing data were addressed.	**nut-12.3** Report any adjustments for measurement error, i.e,. from a validity or calibration study.	
		(d) Cohort study—If applicable, explain how loss to follow-up was addressed.		
		Case-control study—If applicable, explain how matching of cases and controls was addressed.		
		Cross-sectional study—If applicable, describe analytical methods taking account of sampling strategy.		
		(e) Describe any sensitivity analyses.		
**Results**				
Participants	13	(a) Report the numbers of individuals at each stage of the study—e.g., numbers potentially eligible, examined for eligibility, confirmed eligible, included	**nut-13** Report the number of individuals excluded based on missing, incomplete or implausible dietary/nutritional data.	**4**

For peer review only

Item	Item nr	STROBE recommendations	Extension for Nutritional Epidemiology studies (STROBE-nut)	Reported on page #
		in the study, completing follow-up, and analyzed.<br><br>(b) Give reasons for non-participation at each stage.<br><br>(c) Consider use of a flow diagram.		
Descriptive data	14	(a) Give characteristics of study participants (e.g., demographic, clinical, social) and information on exposures and potential confounders<br><br>(b) Indicate the number of participants with missing data for each variable of interest<br><br>(c) Cohort study—Summarize follow-up time (e.g., average and total amount)	**nut-14** Give the distribution of participant characteristics across the exposure variables if applicable. Specify if food consumption of total population or consumers only were used to obtain results.	**Table 1, p5**
Outcome data	15	Cohort study—Report numbers of outcome events or summary measures over time.<br><br>Case-control study—Report numbers in each exposure category, or summary measures of exposure.<br><br>Cross-sectional study—Report numbers of outcome events or summary measures.		
Main results	16	(a) Give unadjusted estimates and, if applicable, confounder-adjusted estimates and their precision (e.g., 95% confidence interval).<br><br>Make clear which confounders were adjusted for and why they were included.<br><br>(b) Report category boundaries when continuous variables were categorized.<br><br>(c) If relevant, consider translating estimates of relative risk into absolute risk for a meaningful time period.	**nut-16** Specify if nutrient intakes are reported with or without inclusion of dietary supplement intake, if applicable.	**NA**
Other analyses	17	Report other analyses done—e.g., analyses of subgroups and interactions and sensitivity analyses.	**nut-17**Report any sensitivity analysis (e.g., exclusion of misreporters or	**NA**

For peer review only

Item	Item nr	STROBE recommendations	Extension for Nutritional Epidemiology studies (STROBE-nut)	Reported on page #
			outliers) and data imputation, if applicable.	
**Discussion**				
Key results	18	Summarize key results with reference to study objectives.		7
Limitation	19	Discuss limitations of the study, taking into account sources of potential bias or imprecision. Discuss both direction and magnitude of any potential bias.	**nut-19** Describe the main limitations of the data sources and assessment methods used and implications for the interpretation of the findings.	**8**
Interpretation	20	Give a cautious overall interpretation of results considering objectives, limitations, multiplicity of analyses, results from similar studies, and other relevant evidence.	**nut-20** Report the nutritional relevance of the findings, given the complexity of diet or nutrition as an exposure.	**8-10**
Generalizability	21	Discuss the generalizability (external validity) of the study results.		10
**Other information**				
Funding	22	Give the source of funding and the role of the funders for the present study and, if applicable, for the original study on which the present article is based.		**16**
Ethics			**nut-22.1**Describe the procedure for consent and study approval from ethics committee(s).	**16**
Supplementary material			**nut-22.2** Provide data collection tools and data as online material or explain how they can be accessed.	**Appendix File**

For peer review only
